# Selective and Adjustable Removal of Phenolic Compounds from Water by Biquaternary Ammonium Polyacrylonitrile Fibers

**DOI:** 10.1021/acsomega.1c02048

**Published:** 2021-07-16

**Authors:** Jingjing Feng, Jiaoru Ran, Minli Tao, Wenqin Zhang

**Affiliations:** † Department of Chemistry, School of Sciences, Tianjin University, Tianjin 300072, P. R. China; ‡ National Demonstration Center for Experimental Chemistry & Chemical Engineering Education, Tianjin University, Tianjin 300350, P. R. China

## Abstract

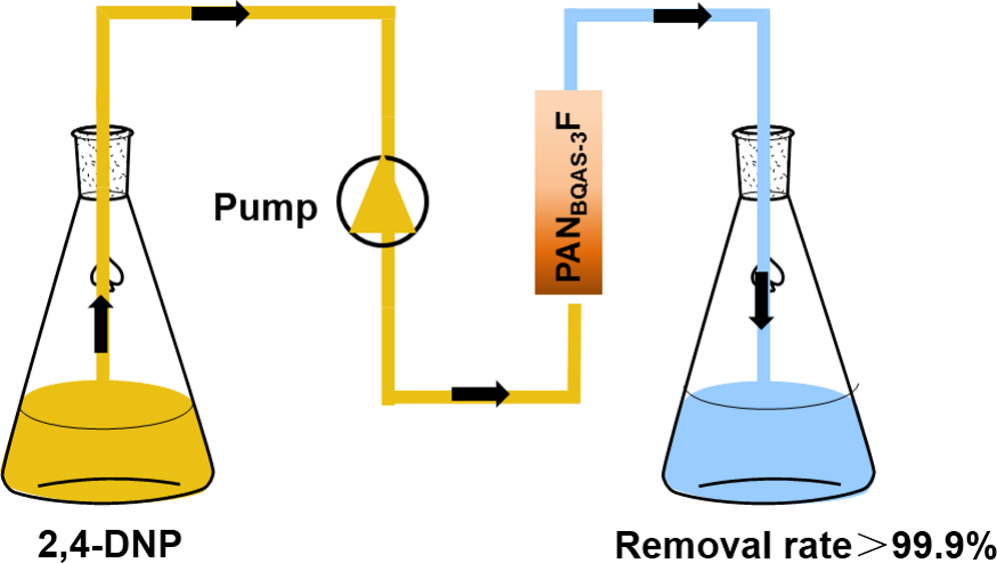

A series of biquaternary ammonium-functionalized fibers were developed to efficiently realize selective removal of phenolic compounds from water. Fourier transform infrared spectroscopy and X-ray photoelectron spectroscopy were employed to determine the successful preparation of functionalized fibers. Scanning electron microscopy, X-ray diffraction (XRD) patterns, and elemental analysis were used to analyze the microstructure and composition. First, the adsorption result shows that a fiber with a three-carbon alkyl chain (PAN_BQAS-3_F) has the maximum adsorption capacity for 2,4-dinitrophenol (2,4-DNP) (406 mg g^–1^). Electrostatic attraction and π–π interaction are the main forces in adsorption. The adsorption kinetics studies display that PAN_BQAS-3_F can rapidly adsorb 2,4-DNP in 10 min and achieve equilibrium within 20 min. The adsorption process of 2,4-DNP by PAN_BQAS-3_F follows the Langmuir model, demonstrating that the process is more consistent with monolayer adsorption. What is more, the adsorbent PAN_BQAS-3_F can be reused after 10 adsorption/desorption cycles and still maintains an excellent removal rate (99%). Otherwise, PAN_BQAS-3_F was used in a continuous flow process and exhibited a removal rate of more than 96%, which certifies that PAN_BQAS-3_F is an excellent adsorbent and can be utilized in practice.

## 1 Introduction

2,4-Dinitrophenol (2,4-DNP) is widely used in industries processes, such as dyes, insecticides, preservatives, explosives, and paper bleaching.^1^ Therefore, a great deal of 2,4-DNP wastewater is produced in the manufacturing process and trace 2,4-DNP is also contained by all of abovementioned products. Due to the low biodegradability, high solubility, and stability, 2,4-DNP has become a typical pollutant and has been banned by the American Environmental Protection Agency and the European Union. In particular, the ingestion of 2,4-DNP may cause harmful effects on human health, like skin allergy, cardiovascular diseases, and low concentration carcinogenesis to human health.^2^ Therefore, it is significant to develop efficient methods for the removal of 2,4-DNP from wastewater before being discharged to the environment.

To achieve this purpose, many methods such as photocatalysis,^3,4^ adsorption,^5−7^ electrochemical degradation,^8,9^ and biochemical reactions^10^ have been explored. Among these approaches, adsorption methods have aroused considerable interest owing to their simplicity, efficiency, and low cost.^11^ Till now, various adsorbents including silica gels,^5,12^ metal–organic frameworks,^6^ and activated carbon^13,14^ have been used to remove 2,4-DNP from an aqueous solution. However, some of these adsorbents have the disadvantages of high cost, a tedious preparation process, and unsatisfactory recyclability, which hinder their practical applications. Thus, there still remains a great challenge to develop effective adsorption materials to overcome these defects.

A polyacrylonitrile fiber (PANF) is a low-cost material with a large surface and high mechanical strength and contains numerous nitrile groups, which can be easily transformed into various functionalized moieties.^15,16^ To date, many kinds of functionalized polyacrylonitrile fibers have been prepared and employed to remove organic pollutants and to absorb and recognize metal.^17−22^ However, there are few reports on the application of functionalized PANF on the removal of 2,4-DNP from the aqueous solutions, so it is of great significance and challenge to prepare efficient fiber absorbents for 2,4-DNP removal.

## 2 Results and Discussion

### 2.1 Synthesis and Characterization of Functionalized Fibers

The modification degree of functionalized fibers can be expressed by weight gain (%) and functionality (mmol g^–1^). The corresponding expressions are eq 1(23,24) and eq 2,^25^ respectively
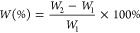
1
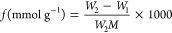
2where *W*_1_ and *W*_2_ represent the weight of before and after modification of the fiber, respectively, and *M* is the molecular weight caused by functional organic molecules (for example, in the preparation of PAN_P_F, *M* is 103 g mol^–1^). Table 1 shows the weight gain and corresponding functionality of different functionalized fibers.

**Table 1:** Modification Degree of Different Functionalized Fibers

entry	fibers	weight gain (%)	functionality (mmol g^–1^)
1	PAN_P_F	24.5	1.91
2	PAN_QAS-1_F	38.6	1.63
3	PAN_QAS-2_F	24.1	1.79
4	PAN_BQAS-2_F	54.0	1.08
5	PAN_BQAS-3_F	73.6	1.21
6	PAN_BQAS-4_F	62.8	1.10
7	PAN_BQAS-5_F	64.2	1.07
8	PAN_BQAS-6_F	40.3	0.76

### 2.2 Characterization of Functionalized Fibers

#### 2.2.1 Fourier Transform Infrared Spectroscopy (FTIR)

**Figure 1:**
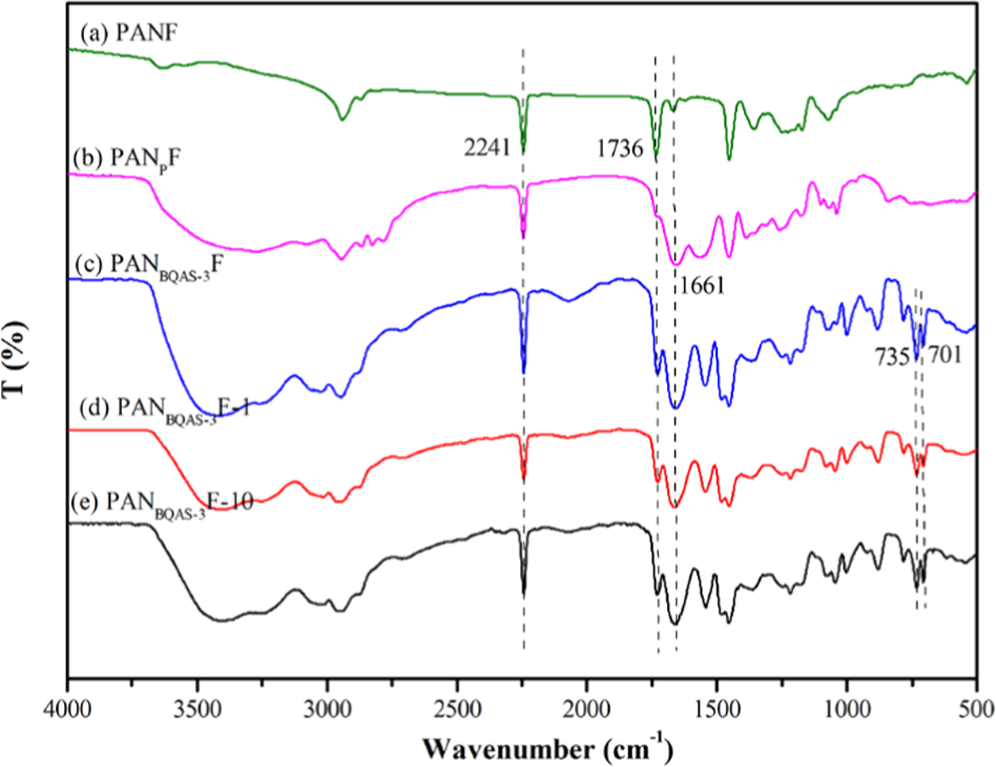
FTIR spectra of (a) PANF, (b) PAN_P_F, (c) PAN_BQAS-3_F, (d) PAN_BQAS-3_F-1, and (e) PAN_BQAS-3_F-10.

The PANF, PAN_P_F, PAN_BQAS-3_F, PAN_BQAS-3_F-1 (fiber recycled once), and PAN_BQAS-3_F-10 (fiber recycled 10 times) samples were pulverized by cutting and then prepared into KBr pellets. The FTIR spectra are presented in Figure 1. The FTIR spectra of PANF (Figure 1a) show that there are two obvious peaks at 2241 and 1736 cm^–1^, corresponding to the stretching vibrations of C≡N and C=O,^26^ which are the characteristic peaks of polyacrylonitrile fiber C≡N and C=O in methyl methacrylate or methyl acrylate, respectively. After modification, PAN_P_F (Figure 1b) and PAN_BQAS-3_F (Figure 1c) showed a wide absorption band between 3600 and 3100 cm^–1^, which corresponds to N–H stretching vibrations. This indicates that *N*,*N*-dimethyl-1,3-propanediamine was successfully grafted onto the fiber surface. Compared to PANF (Figure 1a) and PAN_P_F (Figure 1b), the absorption peak at 1736 cm^–1^ shifted to 1661 cm^–1^, which further indicated that the ester group on the surface of the fiber was transformed into an amide group with higher stability. In addition, PAN_BQAS-3_F shows new peaks at 735 and 701 cm^–1^, which are the stretching vibration peak and the out-of-plane vibration peak of C–H on a benzene ring, respectively, proving the successful immobilization of small molecules of a quaternary ammonium salt. After thePAN_BQAS-3_F was used 10 times, the FTIR spectra of PAN_BQA-3_F-10 (Figure 1d) are basically unchanged compared with those of PAN_BQAS-3_F, which proves that the functionalized fiber is quite stable.

#### 2.2.2 Elemental Analysis (EA)

The elemental analysis (EA) was used to demonstrate the successful preparation of PAN_BQAS-3_F and the stability during the application process. The elemental analysis data of PANF, PAN_P_F, PAN_BQAS-3_F, PAN_BQAS-3_F-1, and PAN_BQAS-3_F-10 are shown in Table 2. Compared with PANF, the content of C and N in PAN_P_F (Table 2, entry 2) decreased and the content of H increased. This is due to the introduction of *N*,*N*-dimethyl-1,3-propanediamine, which contains less carbon and nitrogen and more hydrogen than PANF. After quaternary ammonium salt modification, caused by the introduction of Br atoms in the small molecules, the contents of C, H, and N in PAN_BQAS-3_F decreased compared with PAN_P_F, and the sum of C, H, and N elements (71.4%) decreased significantly (Table 2, entry 3). It is worth mentioning that the elemental analysis data (Table 2, entries 4–5) of PAN_BQAS-3_F reused once and reused 10 times have no significant changes compared with original PAN_BQAS-3_F, which indicates that the fiber has high stability and reusability.

**Table 2:** Elemental Analysis Data

entry	fiber	C (%)	H (%)	N (%)
1	PANF	66.15	5.74	24.29
2	PAN_P_F	53.90	6.83	15.64
3^a^	PAN_BQAS-3_F	51.09	6.79	13.52
4	PAN_BQAS-3_F-1	51.00	6.76	13.64
5	PAN_BQAS-3_F-10	51.31	6.73	13.75

a Weight gain of PAN_BQAS-3_F is 73.6%.

#### 2.2.3 X-ray Photoelectron Spectroscopy (XPS)

**Figure 2:**
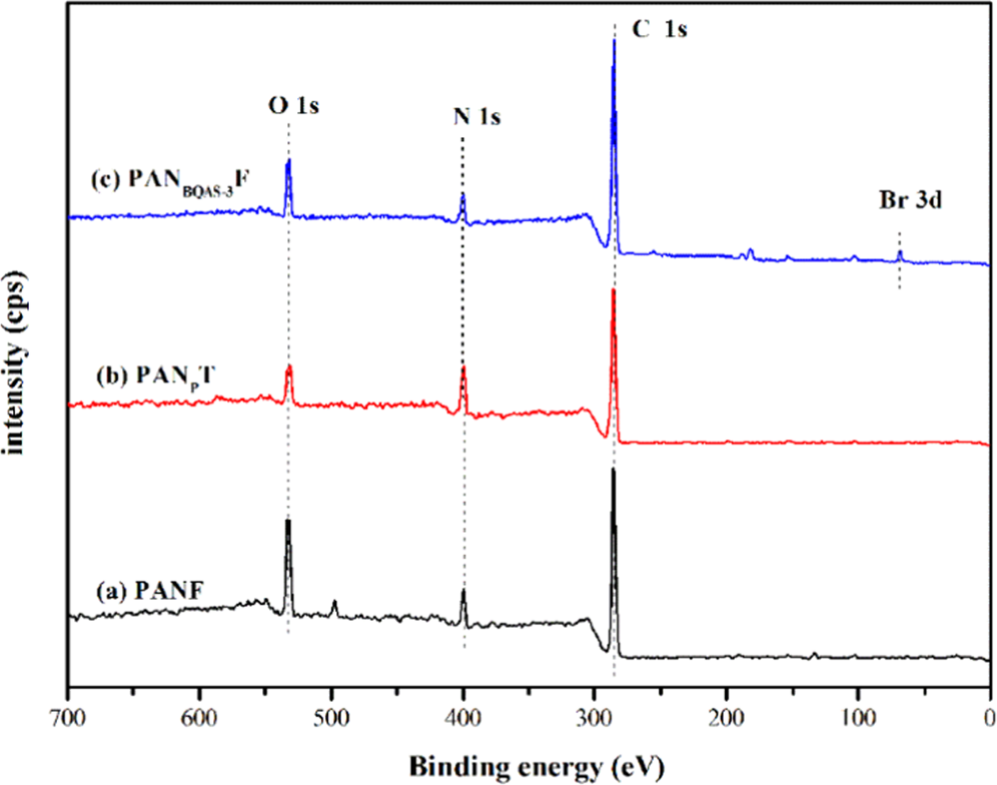
XPS full-scan spectra of (a) PANF, (b) PAN_P_F, and (c) PAN_BQAS-3_F.

To prove the successful functionalization, the chemical composition of the original fiber and the modified fiber were analyzed by XPS. As shown in Figure 2, the XPS full-scan spectra of PANF, PAN_P_F, and PAN_BQAS-3_F show three peaks at 530.89, 397.96, and 284.38 eV, corresponding to the characteristic peaks of O 1s, N 1s, and C 1s, respectively. In addition, a new peak of PAN_BQAS-3_F appears at 68.56 eV,^27^ attributed to the characteristic peak of Br 3d, which indicates that small molecules of bromine-containing quaternary ammonium salt were successfully grafted onto a tertiary amine-functionalized fiber.

**Figure 3:**
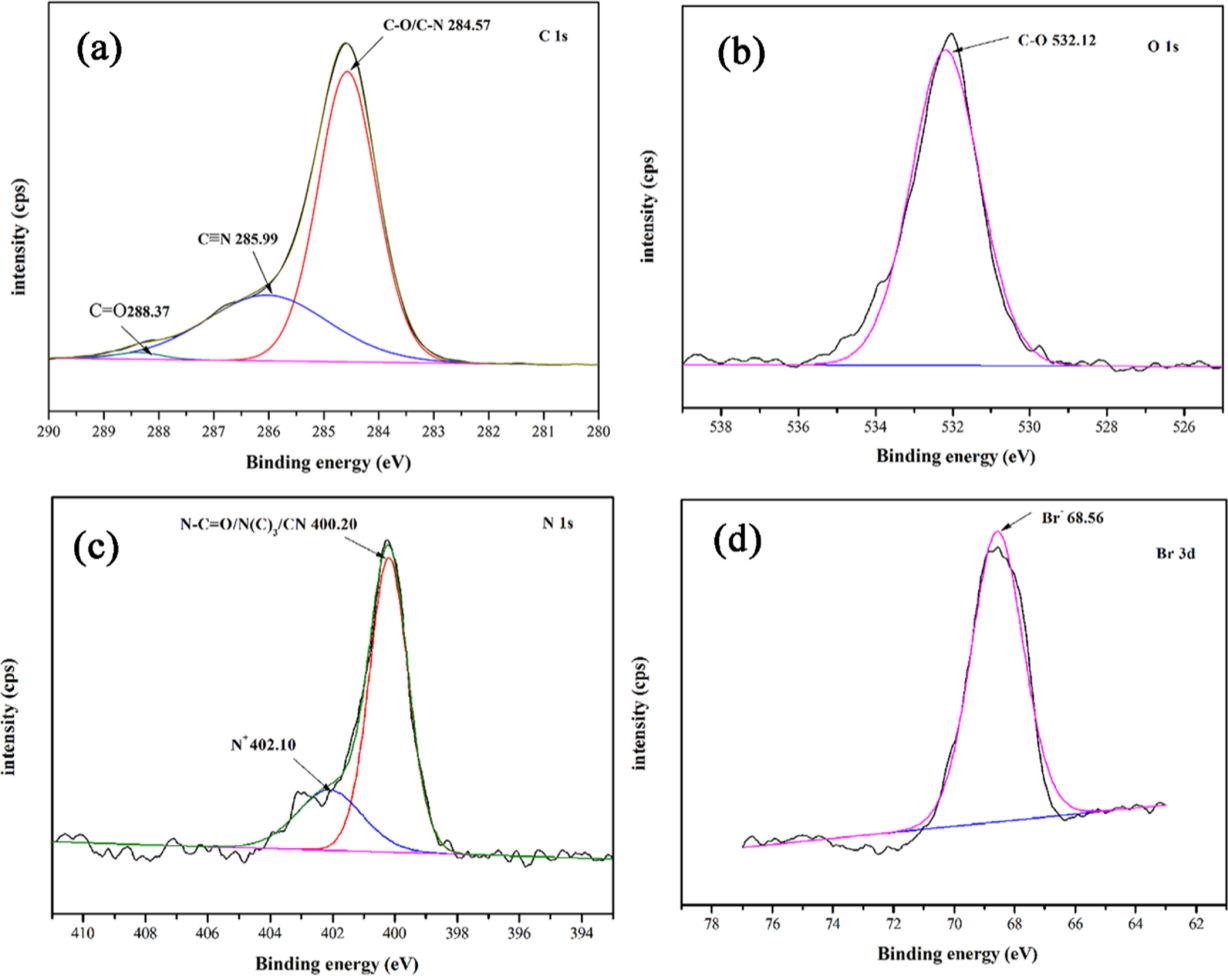
High-resolution XPS spectra of (a) C 1s, (b) O 1s, (c) N 1s, and (d) Br 3d of the PAN_BQAS-3_F.

The XPS high-resolution spectra of C 1s, O 1s, N 1s, and Br 3D of PAN_BQAS-3_F are shown in Figure 3. The three groups of peaks in the C 1s high-resolution spectrum of PAN_BQAS-3_F show three absorption peaks corresponding to the functionalized groups C–O/C–N (284.57 eV), C≡N (285.99 eV), and C=O (288.37 eV) (Figure 3a).^28,29^ In the N 1s high-resolution spectrum of PAN_BQAS-3_F, the binding energies are 400.20 and 402.10 eV belonging to N–C=O/N (C)_3_ and N^+^ groups, respectively,^30^ which further proves the successful grafting of a quaternary ammonium-functionalized fiber (Figure 3c).

#### 2.2.4 Mechanical Strength

Mechanical strength is one of the important indicators to measure the physical stability of the fiber. The results in Table 3 show that PANF (10.90 cN) has high mechanical strength; the strength of PAN_P_F (9.15 cN, modified by *N,N-*dimethyl-1,3-propanediamine) is slightly lower than that of PANF. The mechanical strength of PAN_BQAS-3_F still retains 71% of the fracture strength of PANF. In conclusion, the strength of the fiber is slightly reduced during the modification, but the mechanical strength is still retained high. Moreover, the adsorption operation is generally carried out at room temperature; the adsorption process is mild and the damage to the fiber is minimal (Table 3, entries 4 and 5). The mechanical strength loss of PAN_BQAS-3_F is less and still has high stability after 10 adsorption/analytical cycles, indicating that PAN_BQAS-3_F has high stability and excellent recyclability.

**Table 3:** Mechanical Properties of Different Fibers

entry	fiber	BS (cN)	RBS (%)^a^
1	PANF	10.90	100
2	PAN_p_F	9.15	84
3	PAN_BQAS-3_F	7.74	71
4	PAN_BQAS-3_F-1	6.81	62
5	PAN_BQAS-3_F-10	6.51	60

a Retention of breaking strength (RBS) based on PANF (10.90 cN).

#### 2.2.5 Scanning Electron Microscopy (SEM)

**Figure 4:**
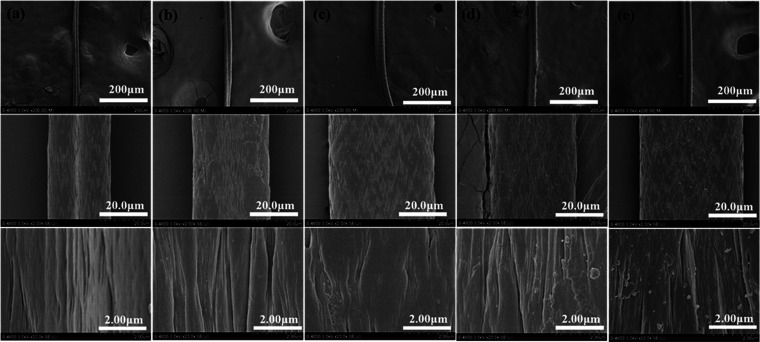
SEM images of (a) PANF, (b) PAN_P_F, (c) PAN_BQAS-3_F, (d) PAN_BQAS-3_F-1, and (e) PAN_BQAS-3_F-10.

The SEM images of PANF, PAN_P_F, PAN_BQAS-3_F, PAN_BQAS-3_F-1, and PAN_BQAS-3_F-10 at different magnifications are presented in Figure 4. Compared with PANF, the surface of PAN_P_F (Figure 4b) and PAN_BQAS-3_F (Figure 4c) was rougher, and their diameters increased gradually with modification. This is because the fiber swells in the process of modification. It is worth mentioning that both the SEM images of PAN_BQAS-3_F-1 and PAN_BQAS-3_F-10 (Figure 4d,e) still maintain the analogous morphology like PAN_BQAS-3_F, which proves the excellent cycling capacity of the PAN_BQAS-3_F.

### 2.3 Adsorption Properties of Functionalized Fibers

The adsorption capacities were calculated by the formulae provided in the Supporting Information.

#### 2.3.1 Adsorption Capacities of Functionalized Fibers

**Figure 5:**
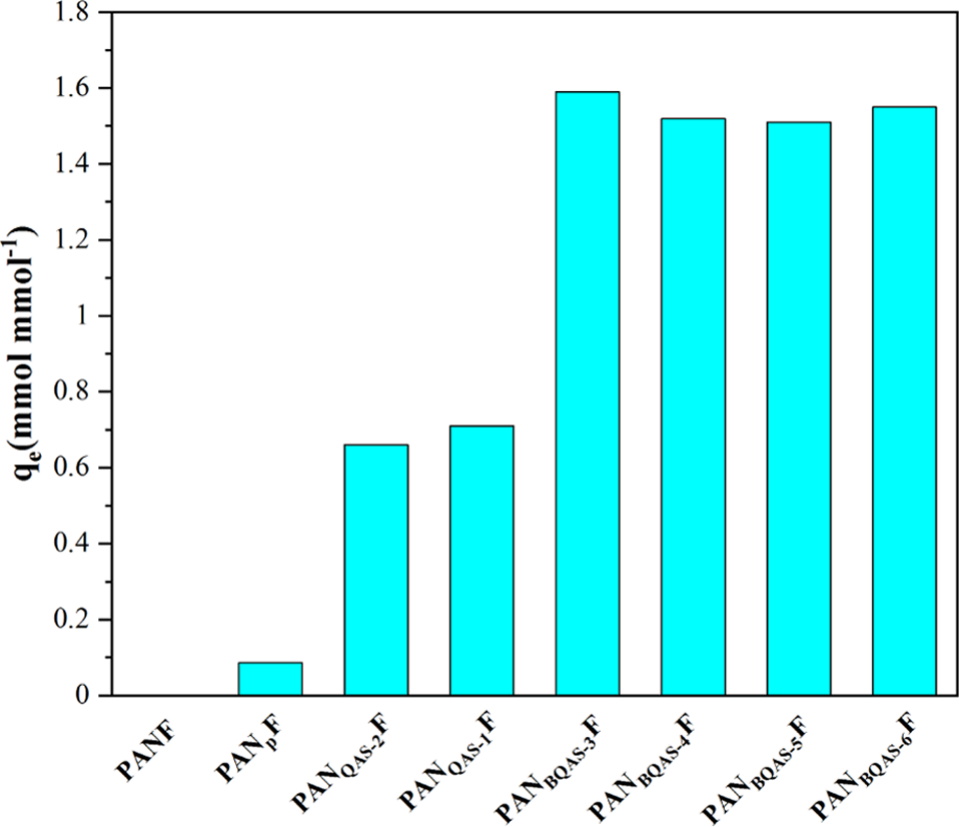
Adsorption of 4-NP by different functionalized fibers.

The 4-NP was selected as a model compound to investigate the adsorption capacities of different functionalized fibers. The adsorption results of 4-NP by various functionalized fibers are shown in Figure 5. The fiber PAN_P_F has a certain adsorption capacity for 4-NP, which may be caused by the hydrogen bond between the amino group of PAN_P_F and 4-NP or the electrostatic attraction between the protonated amino group and 4-NP. For a monoquaternary ammonium-functionalized fiber, the adsorption capacity of PAN_QAS-1_F for 4-NP is slightly higher than PAN_QAS-2_F, which may be due to the π–π interaction of the benzene rings in PAN_QAS-1_F with 4-NP. For a biquaternary ammonium-functionalized fiber, PAN_BQAS-2_F, PAN_BQAS-3_F, PAN_BQAS-4_F, PAN_BQAS-5_F, and PAN_BQAS-6_F all showed good adsorption for 4-NP. Among them, PAN_BQAS-3_F has the best adsorption capacity attributed to the stable six-membered ring structure formed by two nitrogen cations in PAN_BQAS-3_F and phenolic oxygen anions, which can stabilize the transition state better, so PAN_BQAS-3_F shows better adsorption performance. Therefore, in the subsequent experiments, the adsorption properties of PAN_BQAS-3_F were studied systematically.

#### 2.3.2 Adsorption Selectivity of PAN_BQAS-3_F

**Figure 6:**
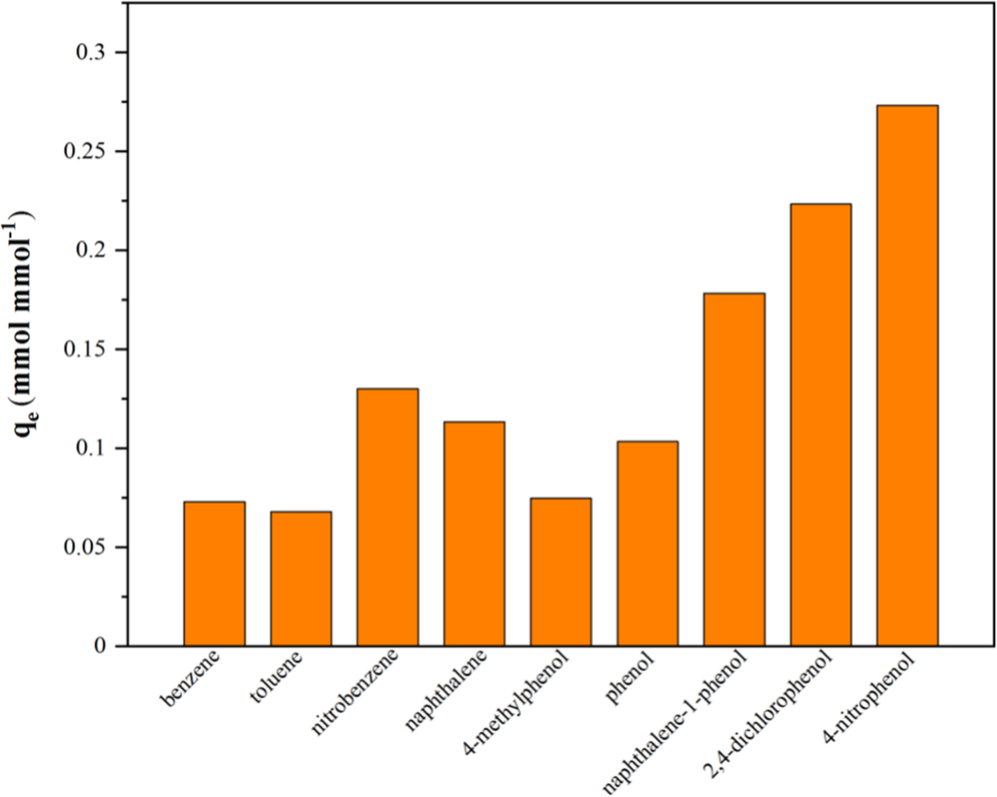
Adsorption selectivity of PAN_BQAS-3_F to different compounds.

Benzene, toluene, nitrobenzene, naphthalene, 4-methylphenol, phenol, naphthalene-1-phenol, 2-dichlorophenol, and 4-NP were selected as target compounds to systematically investigate the adsorption selectivity of PAN_BQAS-3_F for different substances (Table 4). The experimental results are shown in Figure 6. First of all, the adsorption capacity of PAN_BQAS-3_F for phenolic compounds is obviously better than that of aromatic hydrocarbons (phenol > benzene; 4-methylphenol > toluene; 4-NP > nitrobenzene; naphthalene-1-phenol > naphthalene). This is due to the fact that phenolic compounds can be dissociated into phenolic oxygen anions in an aqueous solution and can bind to the positive ion adsorption sites on biquaternary ammonium-functionalized fibers. Second, the adsorption capacity of PAN_BQAS-3_F for phenolic compounds increases with acidity enhancement of these compounds (4-NP > 2,4-dichlorophenol > naphthalene-1-phenol > phenol > 4-methylphenol). This is because the higher the acidity, the easier it is for compounds to ionize hydrogen ions to form phenoxy anions in an aqueous solution, and the stronger the electrostatic attraction is to show the better adsorption capacity. In addition, comparing the adsorption ability of PAN_BQAS-3_F to different aromatic compounds, the adsorption amount of naphthalene is greater than benzene and naphthalene-1-phenol is greater than phenol, which indicates that there is a π–π interaction between the functionalized fiber and the target compound.

**Table 4:**
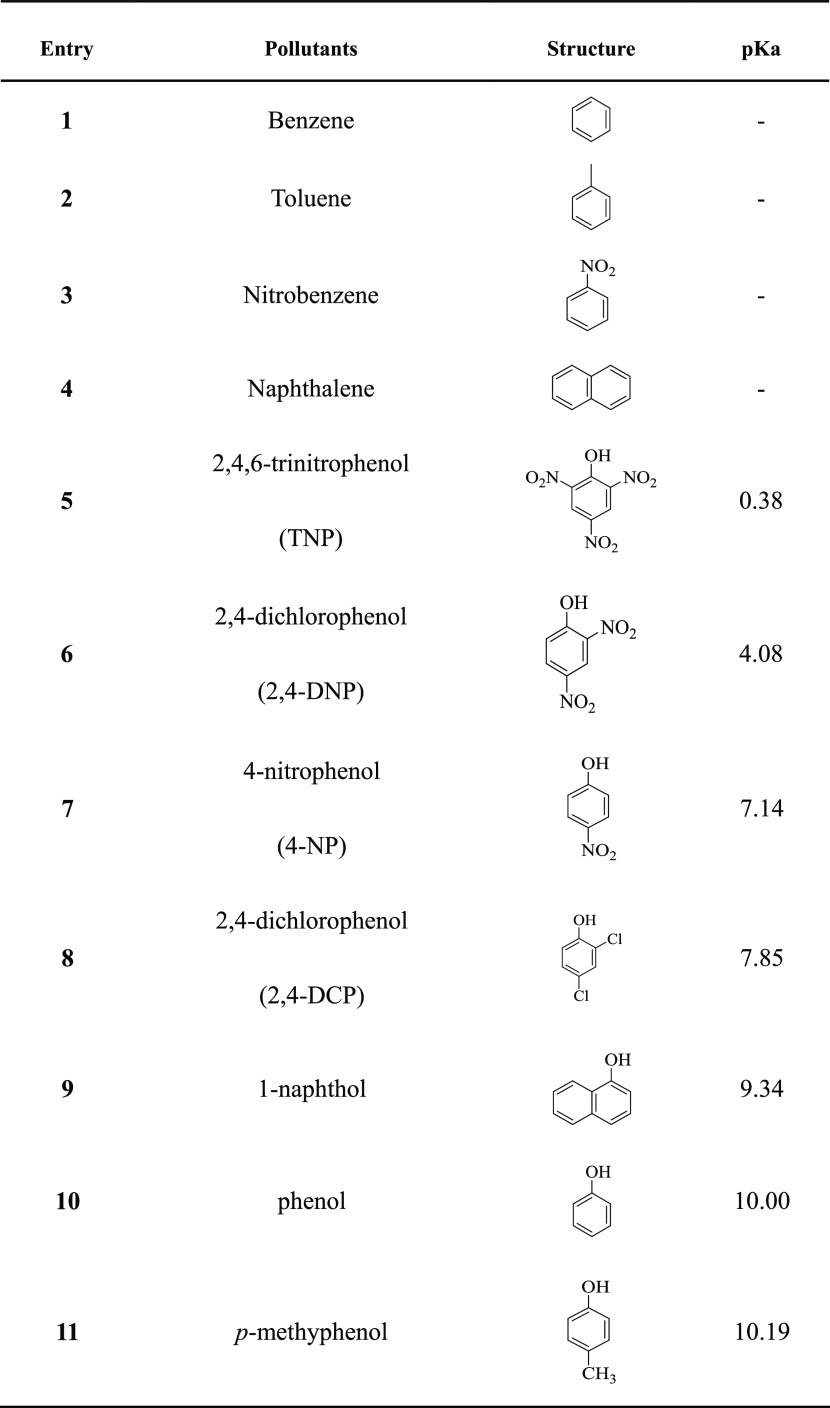
Compounds Used in Adsorption Selectivity of PAN_BQAS-3_F

**Figure 7:**
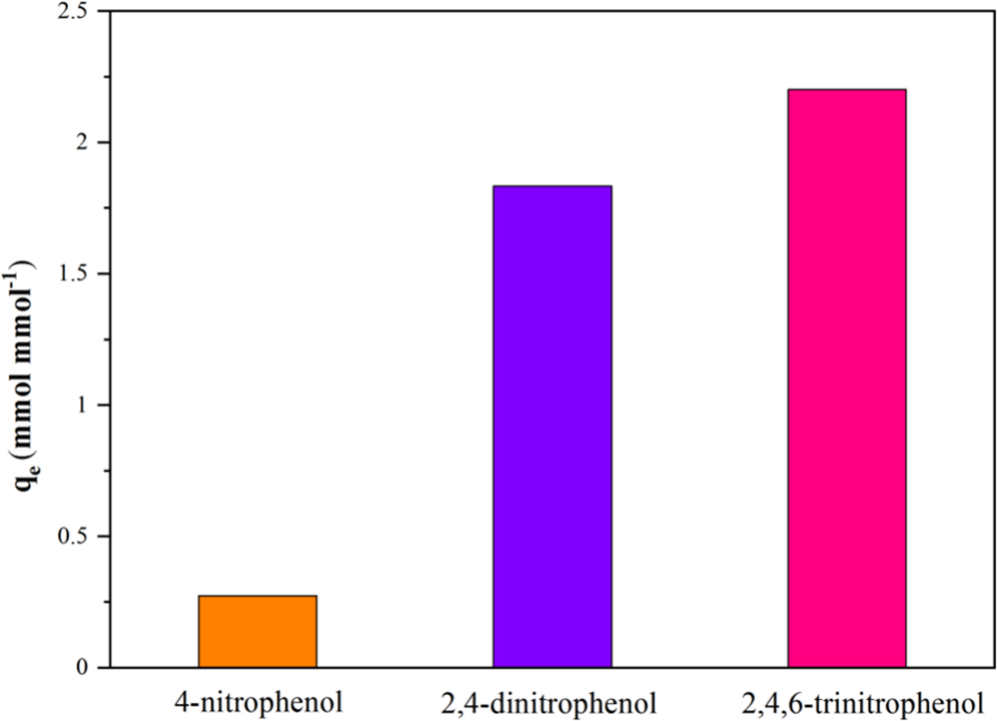
Adsorption capacity of PAN_BQAS-3_F to phenolic compounds with different p*K*_a_s.

From the abovementioned experiment, it can be seen that the acidity or the p*K*_a_ of the compound may be the main factor affecting the adsorption of the functionalized fiber to the target compound. So more acidic phenolic compounds 2,4-DNP (p*K*_a_ = 4.08) and 2,4,6-dinitrophenol (p*K*_a_ = 0.38), were used to explore the adsorption mechanism of functionalized fibers. The results are shown in Figure 7. As expected, the adsorption of phenolic compounds by PAN_BQAS-3_F increases with a decrease in p*K*_a_, which illustrates that the electrostatic attraction of positive and negative charges is the main force of adsorption. In the follow-up research, 2,4-DNP, which has a larger adsorption capacity and is relatively easy to obtain, was selected as the target compound.

#### 2.3.3 Regulating the Adsorption Selectivity of PAN_BQAS-3_F by pH

**Figure 8:**
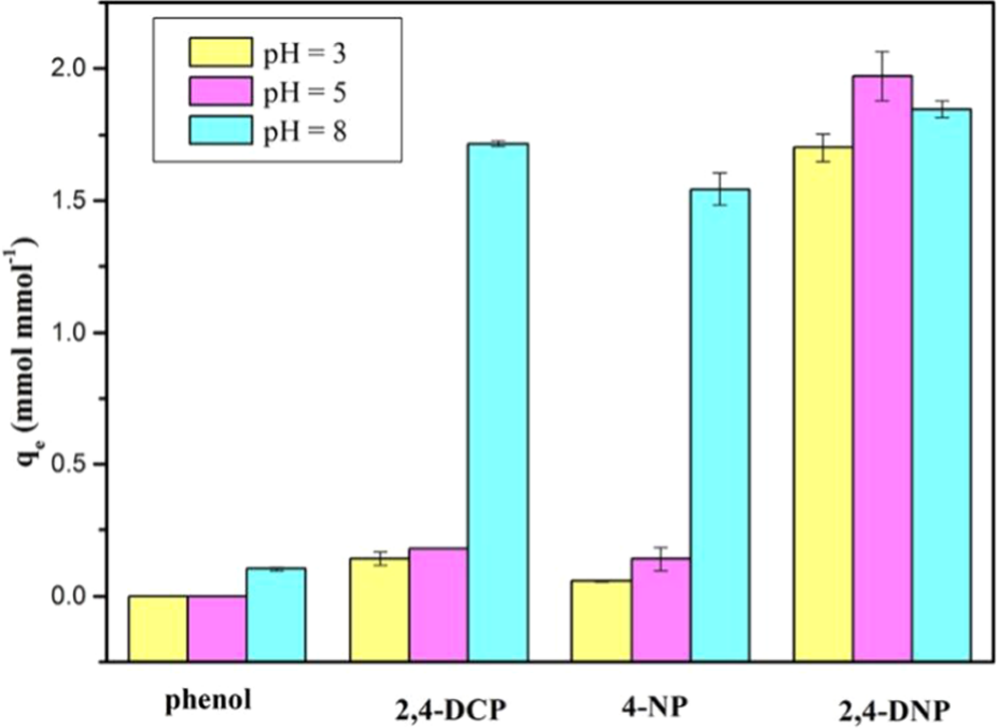
Adsorption capacity of PAN_BQAS-3_F for various phenols under different pH values.

In the paper, adsorption properties of PAN_BQAS-3_F to phenolic compounds were investigated at different pH values, and the results are shown in Figure 8. The adsorption selectivity of the fiber PAN_BQAS-3_F for phenolic compounds can be regulated by changing the pH values of the solution, which determine the existing forms of phenolic compounds in water. First, PAN_BQAS-3_F has better adsorption capacity for 2,4-DNP than other compounds at different pH values because of the strong acidity and a relatively high degree of dissociation of 2,4-DNP. In both acidic and alkaline conditions, 2,4-DNP exists in the form of a phenoxy anion, which can interact well with functionalized fibers. On the other hand, the acidity of 2,4-dichlorophenol and 4-NP is relatively weak, and the degree of dissociation is also weak under acidic conditions. They exist mostly in a molecular form, and the adsorption performance is poor under acidic conditions. However, negative ions can be formed under alkaline conditions, which enhances the interaction with fibers and improves the adsorption capacity of the fiber. Phenol has the weakest acidity and still exists in the form of a molecule under weak alkaline conditions, so the adsorption properties under different pH values are very weak.

#### 2.3.4 Effect of Weight Gain of PAN_BQAS-3_F on Adsorption Capacity

**Figure 9:**
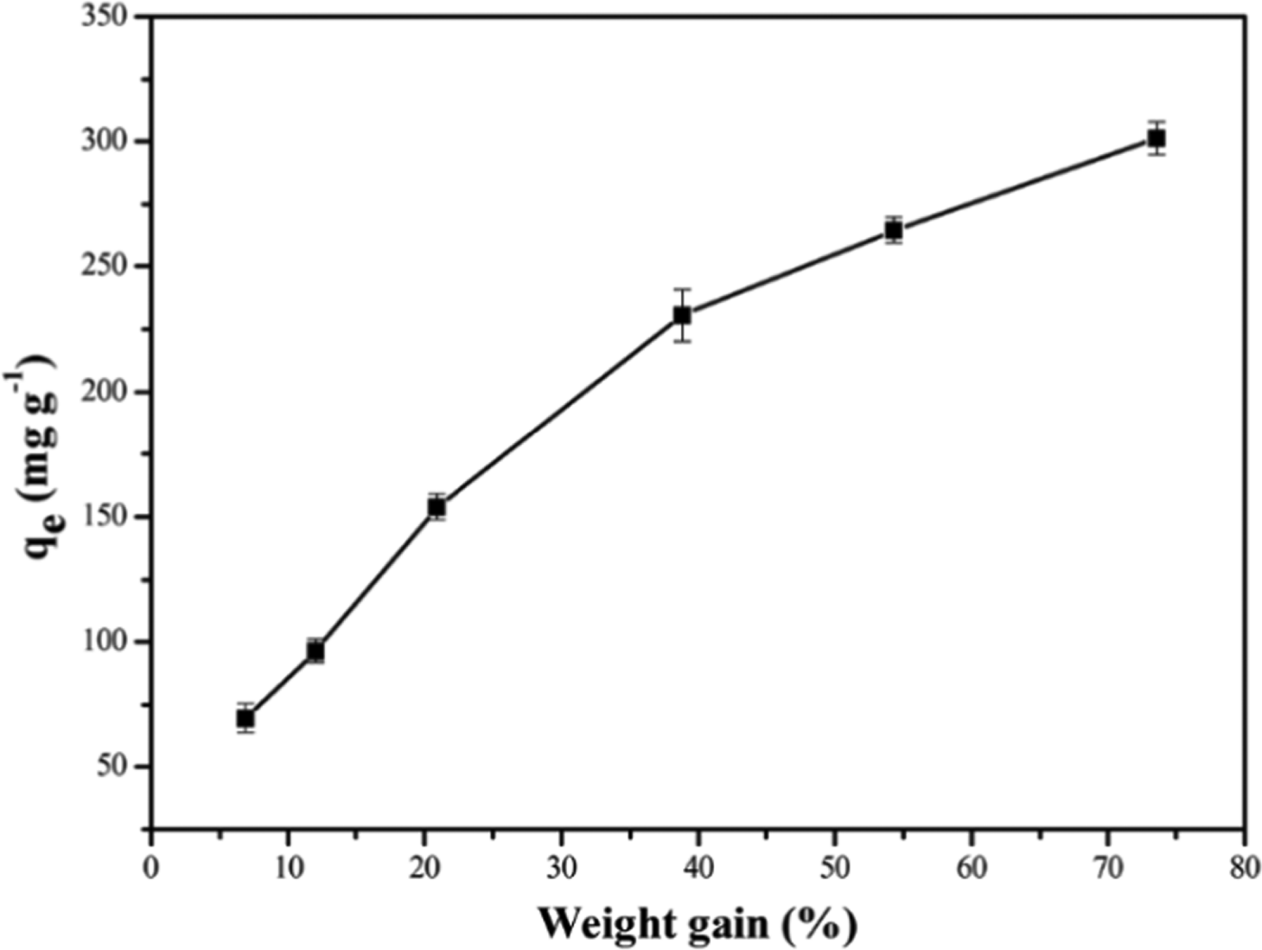
Effect of different weight gains of PAN_BQAS-3_F on adsorption of 2, 4-dinitrophenol.

A series of PAN_BQAS-3_Fs with different weight gains (6.9, 12.1, 20.9, 38.9, 54.3, and 73.6%) were prepared, and their adsorption capacities for 2,4-DNP are shown in Figure 9. With increased weight gain, the adsorption capacity performance was better exhibited. In addition, the mechanical strength tests have shown that the fibers can maintain higher mechanical strength at a greater weight gain (73.6%). Therefore, considering the adsorption capacity and the strength of the functionalized fiber, the PAN_BQAS-3_F fiber with a weight gain of 73.6% was selected for the experiment.

#### 2.3.5 Adsorption Kinetics

**Figure 10:**
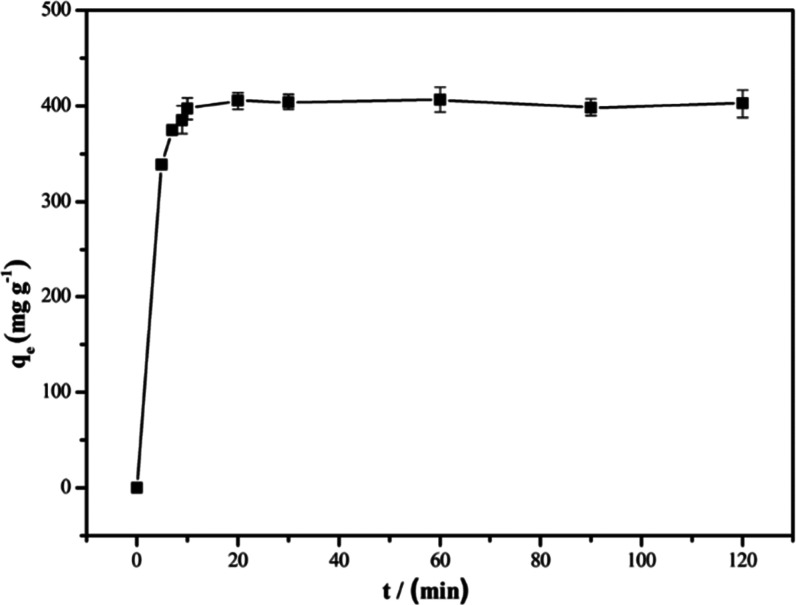
Effect of adsorption time of the adsorption capacity of 2,4-DNP by PAN_BQAS-3_F.

The adsorption kinetics of PAN_BQAS-3_F for 2,4-dinitrophenol was measured, and the results are shown in Figure 10. It can be seen that the adsorption rate of PAN_BQAS-3_F was rapid; it took only 10 min for the fiber to almost reach its saturated adsorption equilibrium (405 mg g^–1^). The adsorption capacity did not further increase after 20 min.

Pseudo-first-order and pseudo-second-order models were used to interpret the kinetics characteristics of the 2,4-DNP adsorption process (Figure S12). The pseudo-first-order eq 3(31) and pseudo-second-order eq 4(32) are given following
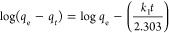
3
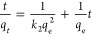
4where *t* (min) is the adsorption time, *q*_e_ (mg g^–1^) is the equilibrium adsorption of the adsorbent, *q*_t_ (mg g^–1^) is the amount of the 2,4-DNP adsorbed by PAN_BQAS-3_F at a given time, and *k*_1_ (min^–1^) and *k*_2_ (g mg^–1^ min^–1^) are pseudo-first-order and pseudo-second-order rate constants, respectively. It can be observed that the *q*_e,exp_ value obtained from the experiment (406.0 mg g^–1^) and the *q*_e,cal_ value calculated based on pseudo-second-order (403.2 mg g^–1^) are more close to each other than that of pseudo-first-order, and the values of *R*^2^ for the pseudo-second-order (0.99989) are greater than the pseudo-first-order model (0.9641), which indicates that the pseudo-second model is more suitable for fitting experimental data. Therefore, the adsorption of 2,4-DNP from aqueous to PAN_BQAS-3_F should be a chemisorption process (Table 5).

**Table 5:** Kinetic Parameters for the Adsorption of 2,4-DNP by PAN_BQAS-3_F

	pseudo-first-order model			pseudo-second-order model		
*q*_e, exp_ (mg g^–1^)	*k*_1_ (min^–1^)	*q*_e,cal_ (mg g^–1^)	*R*^2^	*k*_2_ (g (mg min)^−1^)	*q*_e,cal_ (mg g^–1^)	*R*^2^
406.0	0.259	191.1	0.96481	0.006424	403.2	0.99989

#### 2.3.6 Adsorption Isotherms

**Figure 11:**
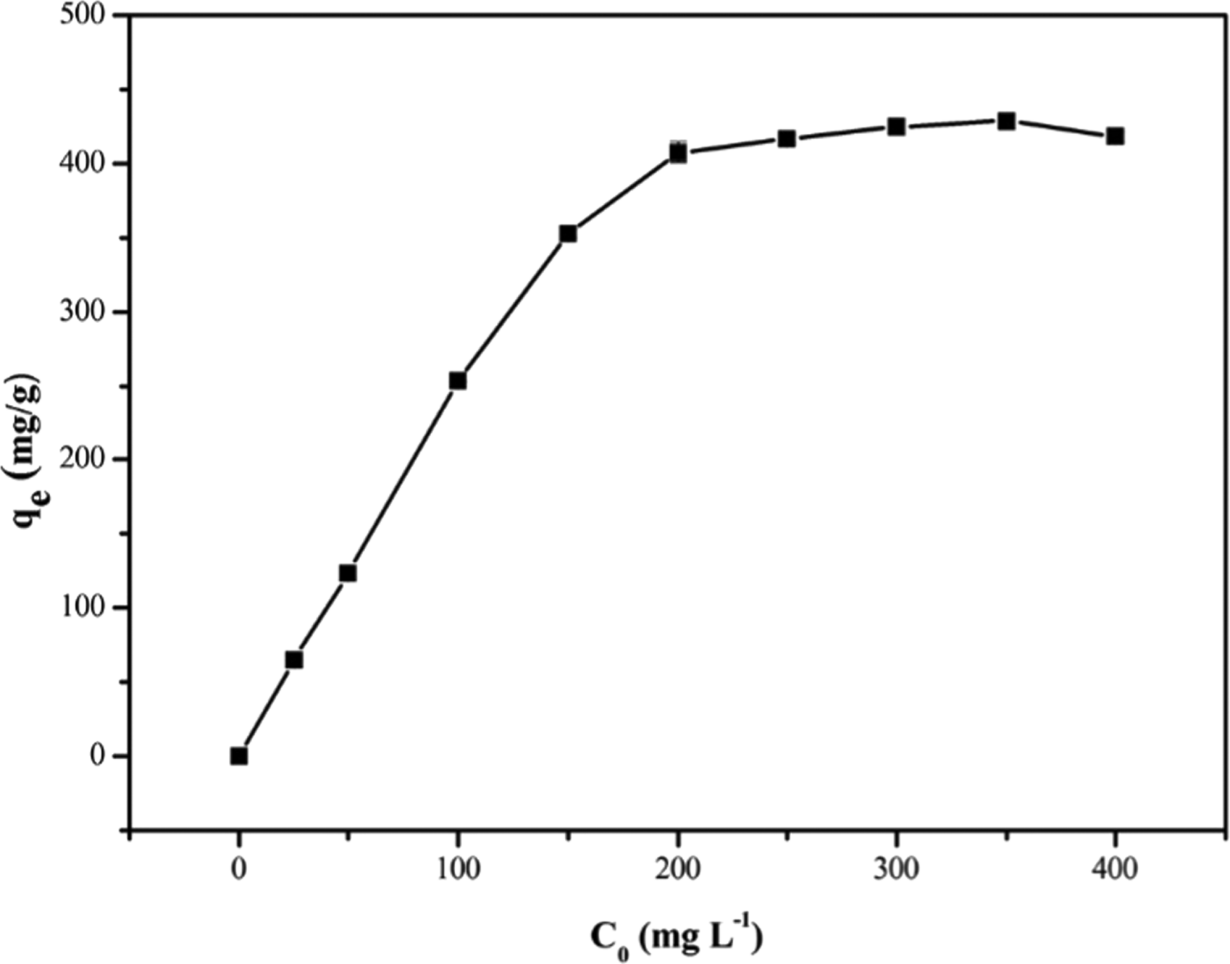
Effect of temperature on the adsorption for 2,4-DNP by PAN_BQAS-3_F.

The effect of the initial concentration of 2,4-DNP on adsorption by PAN_BQAS-3_F was examined (Figure 11). It can be seen that the adsorption amount of PAN_BQAS-3_F to 2,4-DNP gradually increases with an increase in the initial concentration until the adsorption equilibrium is reached. However, when the concentration of 2,4-DNP reached 200 mg L^–1^, the adsorption capacity gradually stabilized.

Langmuir and Freundlich isotherm models were then used to fit the equilibrium adsorption data (Figure S13). The Langmuir isotherm model^33^ assumes that the adsorption process is monolayer adsorption, that there is no interaction between the adsorbates and they are independent, and the surface of the adsorbent is uniform. The Freundlich isotherm model is not only suitable for single molecular layer adsorption but also for multilayer adsorption or uneven surface adsorption. All equations were expressed as^33−36^
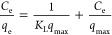
5
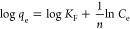
6The^33^ corresponding fitting parameters are listed in Table 6. It can be seen from Table 6 that the *R*^2^ value of the Langmuir model is 0.9992 larger than that of the Freundlich model *R*^2^ (0.7969), indicating that the adsorption process of PAN_BQAS-3_F to 2,4-DNP is more consistent with the Langmuir model and closer to monolayer chemical adsorption. The result shows that the adsorption is due to the interaction of electrostatic attraction and hydrogen bonding. The maximum adsorption capacity (*q*_max_) calculated by the Langmuir isothermal model is 429.0 mg g^–1^, which indicates that PAN_BQAS-3_F has more advantages than other adsorbents (Table S2).

**Table 6:** Parameters for Langmuir and Freundlich Models

model	Langmuir			Freundlich		
equation	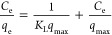			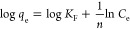		
parameters	*K*_L_ (L mg^–1^)	*q*_max_ (mg g^–1^)	*R*^2^	*K*_F_ ((mg g^–1^)(L mg^–1^)^1/*n*^)	*n*	*R*^2^
value	0.2980	429.0	0.9992	102.87	3.320	0.7969

#### 2.3.7 Adsorption Thermodynamics

**Figure 12:**
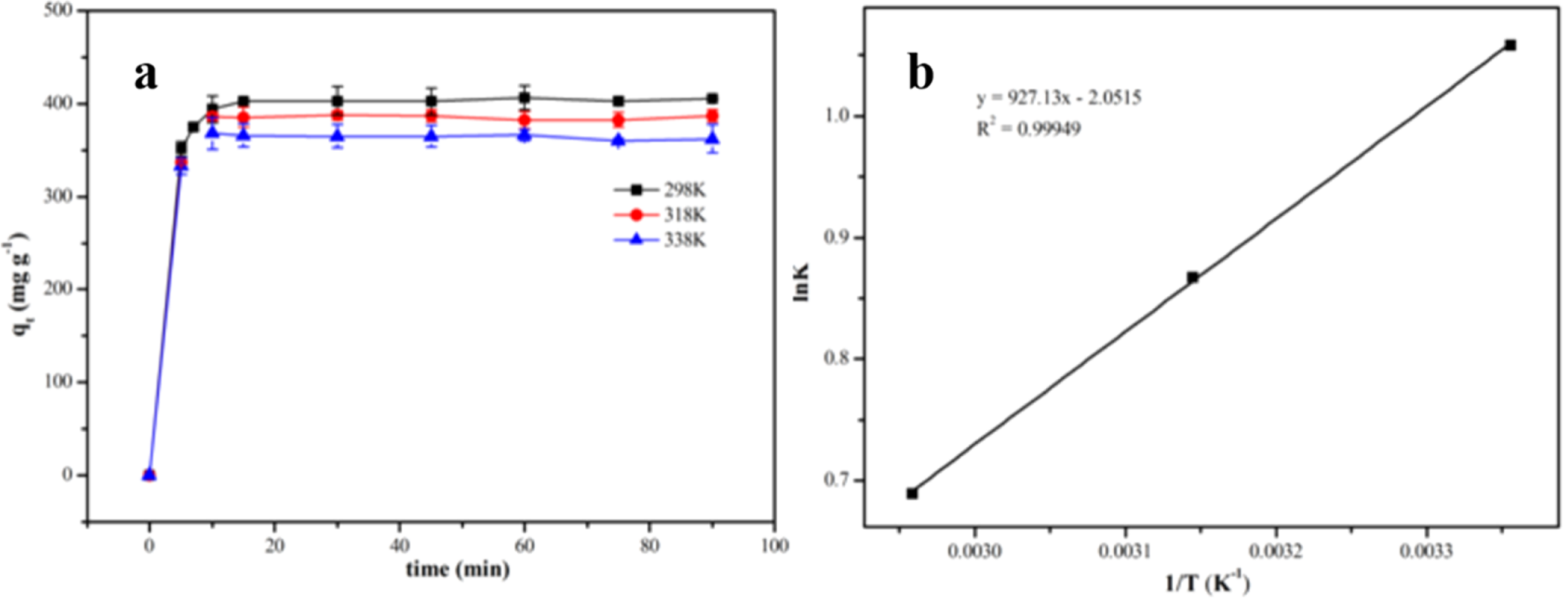
(a) Effect of temperature on the adsorption of 2,4-DNP by PAN_BQAS-3_F. (b) Thermodynamic fitting diagram.

The effect of temperature on the adsorption capacity of 2,4-DNP by PAN_BQAS-3_F was investigated at 298, 318, and 338 K (Figure 12). The adsorption capacities of 2,4-DNP gradually decreased as the temperature increased from 298 to 338 K. Vander Hoff equations provided in the Supporting Information were used to calculate the thermodynamic parameters such as Δ*H*^0^ and Δ*S*^0^ (Table 7).

**Table 7:** Thermodynamic Parameters for the Adsorption of 2,4-Dinitrophenol by PAN_BQAS-3_F

*T* (K)	ln *K*	Δ*H* (kJ mol^–1^)	Δ*S* (J mol^–1^)	Δ*G* (kJ mol^–1^)
298	1.0581			–2.624
318	0.8674	–7.708	–17.06	–2.283
338	0.6897			–1.9417

#### 2.3.8 Desorption and Reusability

**Figure 13:**
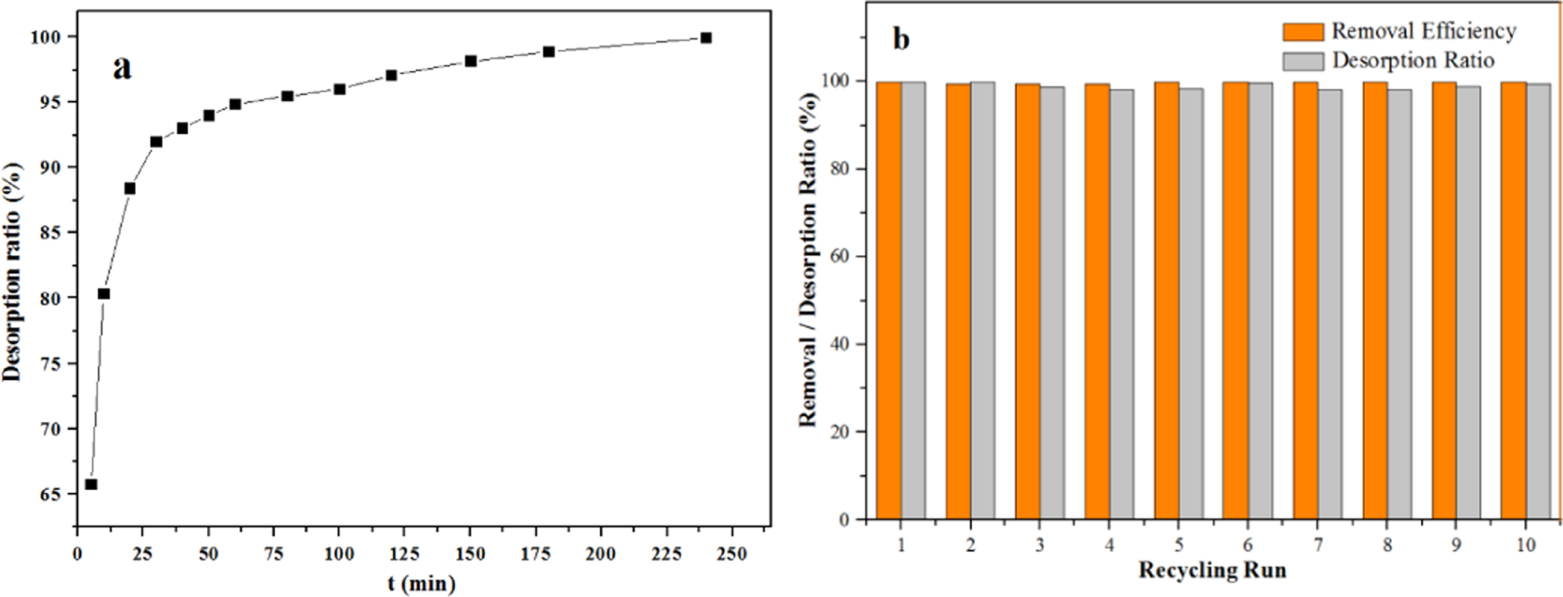
(a) Desorption curve of 2,4-DNP on the PAN_BQAS-3_F fiber. (b) Reusability of PAN_BQAS-3_F in a continuous flow condition.

Recyclability is an important factor of an adsorbent in practical application. The desorption curve of 2,4-DNP from PAN_BQAS-3_F using a NaBr solution as a desorbent is shown in Figure 13a. It is found that the desorption rate reached 91.99% after 30 min. When time increased to 240 min, 2,4-DNP could be completely desorbed from the fiber. Therefore, 240 min was chosen as the optimal desorption time. After 10 times adsorption–desorption cycles (Figure 13b), the adsorption and desorption rate still remained above 99%. The excellent reusability proved the great potential of the PAN_BQAS-3_ adsorbent in real application.

#### 2.3.9 Adsorption Mechanism

**Scheme 1:**
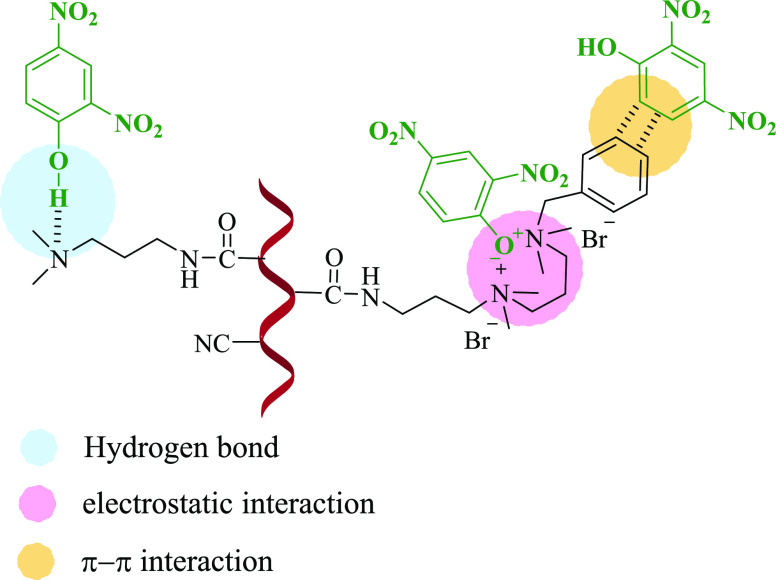
Possible Adsorption Mechanism

Based on the abovementioned experimental results, a possible adsorption mechanism of 2,4-DNP by PAN_BQAS-3_F is proposed and described in Scheme 1. The electrostatic force between a phenoxy anion and a quaternary ammonium group on the fiber is the main adsorption force, so the smaller the p*K*_a_ of the adsorbent, the easier it is to ionize phenoxy, which can enhance adsorption capacity. The electrostatic attraction between phenolic compounds and functionalized fiber increases with the increase in adsorption sites, so the adsorption capacity of biquaternary ammonium-functionalized fibers is better than single quaternary ammonium-functionalized fibers. At the same time, two nitrogen cations in the PAN_BQAS-3_F structure of the biquaternary ammonium-functionalized fiber can synergistically interact with phenoxy anions and form a stable six-membered ring structure, which can better stabilize the transition state, so it has the best adsorption performance. In addition, the π–π interaction between phenolic compounds and the benzene ring on the fiber is also one of the adsorption interaction forces. Therefore, the adsorption is enhanced in the phenolic hydroxyl group, and the nitrogen atom of the residual amino group in the PAN_BQAS-3_F fiber plays an auxiliary role in the adsorption process.

## 3 Conclusions

A series of monoquaternary ammonium- and biquaternary ammonium-functionalized fibers were successfully prepared and used to remove 4-nitrophenol from water. The results showed that the biquaternary ammonium-functionalized fiber (PAN_BQAS-*n*_F, *n* = 2, 3, 4, 5, and 6) exhibited better adsorption of 4-nitrophenol than monoquaternary ammonium-functionalized fiber (PAN_QAS-*n*_F, *n* = 1, 2). Then, PAN_BQAS-3_F was studied to investigate adsorption properties for 2,4-DNP. PAN_BQAS-3_F shows excellent adsorption capacity (406.0 mg g ^–1^) and reusability (removal efficiency >99% after 10 adsorption/desorption cycles). The adsorption experiment data fitted well with the pseudo-second-order kinetic model and the Langmuir adsorption isotherm model, indicating that the adsorption process is chemisorption of a monolayer. In addition, PAN_BQAS-3_F exhibited remarkable and obvious performance under continuous flow conditions, which can be referred to in the video in the Supporting Information.

## 4 Experimental Section

### 4.1 Reagents and Instruments

#### 4.1.1 Reagents

A polyacrylonitrile fiber with a diameter of 20 ± 0.5 μm (purchased from the Fushun Petrochemical Corporation of China) was cut into lengths of 5–10 cm before use. *N,N*-dimethyl-1,3-propanediamine, 1,2-dibromoethane, 1,3-dibromopropane, 1,4-dibromobutane, 1,5-dibromopentane, 1,6-dibromohexane, *N*,*N*-dimethylbenzylamine, benzyl bromide, bromoethane, ethanol, diethyl ether, acetonitrile, phenol, 4-chlorophenol, 4-nitrophenol, and ethyl acetate were all analytical grade and used without further purification. The water was deionized (24.08 μS cm^–1^).

#### 4.1.2 Instruments

An AVATAR360 FTIR spectrometer (Thermo Nicolet) was employed to obtain FTIR spectra of the fibers. Elemental analysis (EA) data of the original and functionalized fibers were obtained using an ElementarVario EL instrument. X-ray photoelectron spectroscopy (XPS) was obtained using a VersaProbe spectrometer (model PHI-5000). X-ray powder diffraction (XRD) patterns were recorded on a D/MAX-2500 X-ray diffractometer (Rigaku Corporation). The thermal stability of fibers was investigated using an STA409PC TGA/DSC simultaneous thermal analyzer (Netzsch company, Germany). The mechanical properties of the different fiber samples were tested using an electronic single fiber strength tester LLY-6 (Laizhou Electronic Instrument Corporation, China). The surface morphology of the fibers was observed using a microscope (Hitachi, model S-4800). The pH values were determined using a pH meter (Model PHS-25). A TU-1901 dual-beam ultraviolet visible spectrophotometer (Persee) was employed to determine the concentrations of various phenol solutions. ^1^H NMR (600 MHz) spectra were recorded on a JEOL JNM ECZ600R instrument using tetramethylsilane as an internal standard. ^1^H NMR (400 MHz) spectra were recorded on a BRUKER-AVANCE III instrument using tetramethylsilane as an internal standard.

### 4.2 Synthesis of the Functionalized Fiber

**Scheme 2:**
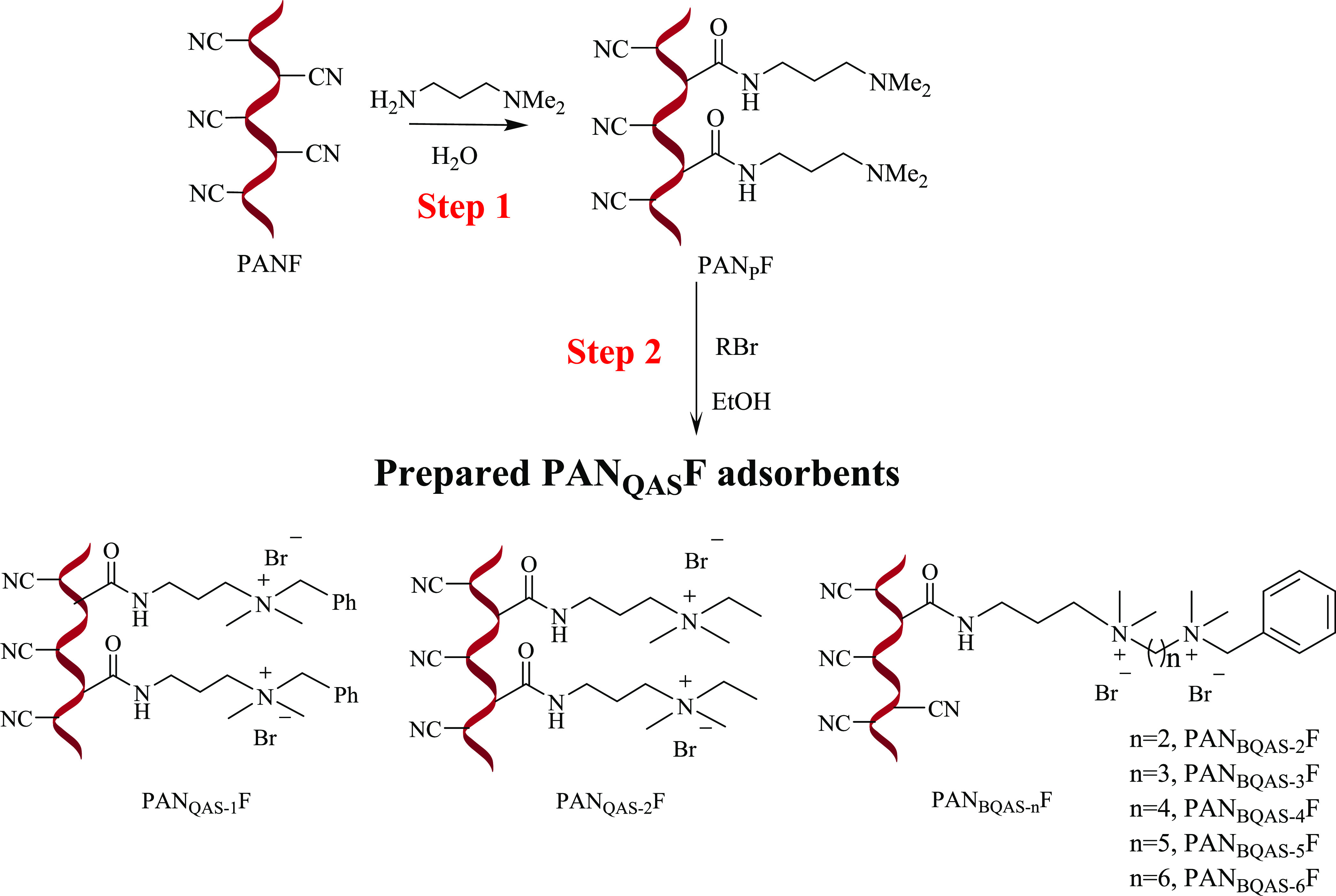
Preparation of the Functionalized Fibers

The quaternary ammonium salt-functionalized fiber PAN_QAS_Fs were prepared through a two-step reaction, as shown in Scheme 2. First, *N,N*-dimethyl-1,3-propanediamine was grafted onto the PANF to obtain the aminated fiber (PAN_P_F). Then, the fiber further reacted with halogenated hydrocarbon through quaternization to get different quaternary ammonium salt-functionalized fibers PAN_QAS_Fs. The detailed preparations of functional organic molecules (RBr) and functionalized fibers are described in the Supporting Information (Scheme S1).

Step 1: Dried PANF (1.0 g), *N*,*N*-dimethyl-1,3-propanediamine (20 mL), and deionized water (10 mL) were added to a three-neck flask. The mixture was stirred and refluxed for 4.5 h. After that, the functionalized fiber was filtered and washed with hot water (60–70 °C) until neutral. The fiber was dried overnight at 60 °C under vacuum to give PAN_p_F (Scheme S2).

Step 2: Dried PAN_P_F (1.00 g), corresponding RBr (2 mmol), and ethanol (20 mL) were added to a three-neck flask. The mixture was stirred and refluxed for 4.0 h. After the reaction, the functionalized fiber was filtered and washed with ethanol in a soxhlet extractor to remove unreacted small molecules. The fiber was dried overnight at 60 °C under vacuum to give PAN_QAS-1_F, PAN_QAS-2_F, and PAN_BQAS-*n*_Fs (Schemes S3 and S4).

### 4.3 Adsorption Experiments

#### 4.3.1 Adsorption Properties of Different Functionalized Fibers for 4-Nitrophenol

Dried functionalized fibers (PAN_P_F, PAN_QAS-1_F, PAN_QAS-2_F, and PAN_BQAS-*n*_Fs) (15 mg) were immersed in 25 mL of 4-nitrophenol (4-NP) (200 mg L^–1^, pH = 8) and stirred for 12 h. The concentrations of 4-NP before and after adsorption were determined by UV–vis spectroscopy.

#### 4.3.2 Adsorption Kinetics, Isotherm, and Thermodynamics

Dried PAN_BQAS-3_F (15 mg) was immersed in 40 mL of a 2,4-DNP solution (200 mg L^–1^, pH = 6) and then the mixture was stirred at required time and temperature. For kinetics research, after being stirred for a certain time at room temperature, the fiber was filtered and the remaining concentration of 2,4-DNP was measured by UV–vis spectroscopy. Similarly, the isotherm and thermodynamic experiments were carried out at different initial concentrations (25–400 mg L^–1^) and temperatures (298, 303, and 308 K), respectively.

#### 4.3.3 Flow Adsorption Experiment

Dried PAN_BQAS-3_F (0.25 g) was filled into a cylindrical tube with a length of 100 mm and a diameter of 5.7 mm, and then the solution of 2,4-DNP with an initial concentration of 200 mg L^–1^ and pH = 6 was pumped through the cylindrical tube with the functionalized fiber at a flow rate of 2 mL min^–1^ using a peristaltic pump, and the liquid was collected. The concentration of 2,4-DNP in the effluent was determined by UV–vis spectroscopy.

#### 4.3.4 Desorption and Reversibility of PAN_BQAS-3_F

Adsorption: First, dried PAN_BQAS-3_F (0.20 g) was filled into a cylindrical tube with a length of 100 mm and a diameter of 5.7 mm, and then a 2,4-DNP solution (*V* = 250 mL, *C*_o_ = 200 mg L^–1^, pH = 6) was pumped through the cylindrical tube containing the functionalized fiber at a flow rate of 2 mL min^–1^, and the effluent was collected. The absorbance of 2,4-DNP in the effluent was determined by UV–vis spectroscopy.

Desorption: A NaBr solution (0.1 mmol L^–1^) was used as an eluent and pumped through a cylindrical tube containing the adsorbed functionalized fiber at a flow rate of 2 mL min^–1^. The liquid was collected in a receiving bottle at the same time interval, and the desorption ratio of the fiber at different times was studied. The concentration of 2,4-DNP in the effluent was determined by UV–vis spectroscopy. Such adsorption/desorption recycling is used to determine the reuse performance of the functionalized fiber.

## Supporting Information Available

Detailed preparation and NMR data of quaternary ammonium salt, synthesis of the functionalized fiber, the standard concentration curve of 2,4-dinitrophenol and 4-nitrophenol, adsorption kinetics and the isotherm model fitting diagram, and comparison of 2,4-dinitrophenol removal performance of PAN_BQAS-3_F with recently reported adsorbents (PDF)
Adsorption-attachment1 (MOV)
Desorption-attachment2 (MOV)

## Supplementary Material

ao1c02048_si_001.pdf

ao1c02048_si_002.mov

ao1c02048_si_003.mov
