# Supplementary material for: Selective and Adjustable Removal of Phenolic Compounds from Water by Biquaternary Ammonium Polyacrylonitrile Fibers
Source: ACS Omega. 2021 Jul 16;6(29):18836–47. doi: 10.1021/acsomega.1c02048 (PMC8320098; doi:10.1021/acsomega.1c02048)
Supplement: Supplementary file 1 — ao1c02048_si_001.pdf [file ao1c02048_si_001.pdf]

SUPPORTING INFORMATION

Selective and adjustable removal of phenolic compounds from water by biquaternary ammonium polyacrylonitrile fiber

Jingjing Feng[a], Jiaoru Ran[a], Minli Tao[, a, b], Wenqin Zhang[a]*

[a]Department of Chemistry, School of Sciences, Tianjin University, Tianjin, 300072, P. R. China.

[b]National Demonstration Center for Experimental Chemistry & Chemical Engineering Education, Tianjin University, Tianjin 300350, China.

Table of contents

1. Preparation of small molecules of quaternary ammonium salt S3

2. Synthesis of the PAN_PF S7

3. Synthesis of the $PAN_{QAS-1}F$ and $PAN_{QAS-2}F$ S7

4. Synthesis of the $PAN_{BQAS-2}F$, $PAN_{BQAS-3}F$, $PAN_{BQAS-4}F$, $PAN_{BQAS-5}F$ and $PAN_{BQAS-6}F$ S8

5. Mixed phenolic adsorption experiment S8

6. Thermogravimetric analysis (TGA) S9

7. XRD patterns S9

8. Effect of $PAN_{BQAS-3}F$ dosage on adsorption capacity S10

9. Effect of solution pH on the adsorption capacity S10

10. Relationship between pH initial and pH final for pHpzc determination S10

11. Standard concentration curve of 2,4-dinitrophenol and 4-nitrophenol S10

12. Pseudo first-order and pseudo second-order kinetics model for the adsorption of 2,4-dinitrophenol by $PAN_{BQAS-3}F$ S11

13. Langmuir and Freundlich isotherm plots for the adsorption of 2,4-dinitrophenol by $PAN_{BQAS-3}F$ S11

14. Breakthrough curves for 2,4-DNP solutions S11

15. Comparison of 2,4-dinitrophenol removal with recently reported adsorbents S12

16. Removal of 2,4-dinitrophenol by $PAN_{BQAS-3}F$ under continuous flow condition S13

17. Desorption of 2,4-dinitrophenol by $PAN_{BQAS-3}F$ under continuous flow condition S13

18. Reference S13

1. Preparation of small molecules of quaternary ammonium salt

According to the preparation method in the reference[1-2], 60 mL acetonitrile dissolved in **compound 2** (2.70g, 0.02 mol) was slowly dripped through a constant pressure drop funnel to a 250 mL three-port flask containing **compound 1** (0.1 mol). The mixture was then stirred at 50 °C for 16 h. After the reaction, the solvent was evaporated and concentrated, and anhydrous ether (100 mL) was added. The solid was placed overnight to give a large amount of white solid. Then the solid was filtered and washed by anhydrous ether until the filtrate was clear to obtain **compound 1a**, **compound 1b**, **compound 1c**, **compound 1d** and **compound 1e**.

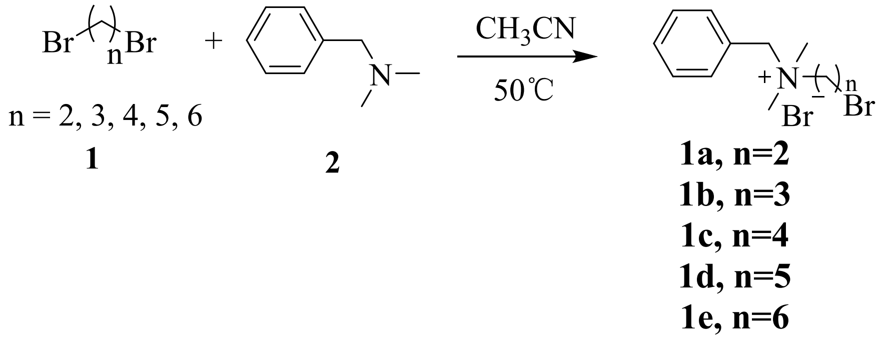

Scheme S1. Synthesis of small molecules of quaternary ammonium salt

compound 1a (86% yield): ^{1}H NMR (600 MHz, DMSO-d_6) δ 7.54 – 7.47 (m, 5H), 4.56 (s, 2H), 3.95 – 3.90 (m, 2H), 3.72 – 3.67 (m, 2H), 2.97 (s, 6H).

compound 1b (86% yield**):** ^{1}H NMR (400 MHz, $CDCl_3$) δ 7.65 (d, J = 7.8 Hz, 2H), 7.45- 7.34 (m, 3H), 4.99 (d, J = 7.4 Hz, 2H), 3.76 (dd, J = 9.8, 6.5 Hz, 2H), 3.47 (t, J = 5.8 Hz, 2H), 3.28 (d, J = 8.6 Hz, 6H), 2.50 (ddd, J = 19.0, 14.8, 10.2 Hz, 2H).

compound 1c (87% yield**):** ^{1}H NMR (400 MHz, D_2O) δ 7.58 (s, 5H), 4.53 (s, 2H), 3.58 (s, 2H), 3.38-3.30 (m, 2H), 3.07 (s, 6H), 1.98 (dd, J = 31.5, 25.0 Hz, 4H).

compound 1d (84% yield)**:** ^{1}H NMR (400 MHz, DMSO-d_6) δ 7.64 – 7.46 (m, 5H), 4.62 (d, J = 3.3 Hz, 2H), 3.59 (t, J = 6.6 Hz, 2H), 3.34 – 3.27 (m, 2H), 3.01 (d, J = 15.3 Hz, 6H), 1.95 – 1.74 (m, 4H), 1.45 – 1.34 (m, 2H).

compound 1e (80% yield)**:** ^{1}H NMR (400 MHz, DMSO-d_6) δ 7.54 (d, J = 8.0 Hz, 5H), 4.58 (d, J = 18.6 Hz, 2H), 3.55 (t, J = 6.5 Hz, 2H), 3.34 – 3.21 (m, 2H), 2.97 (d, J = 11.4 Hz, 6H), 1.89 – 1.73 (m, 4H), 1.51 – 1.39 (m, 2H), 1.30 (dd, J = 14.4, 7.3 Hz, 2H).

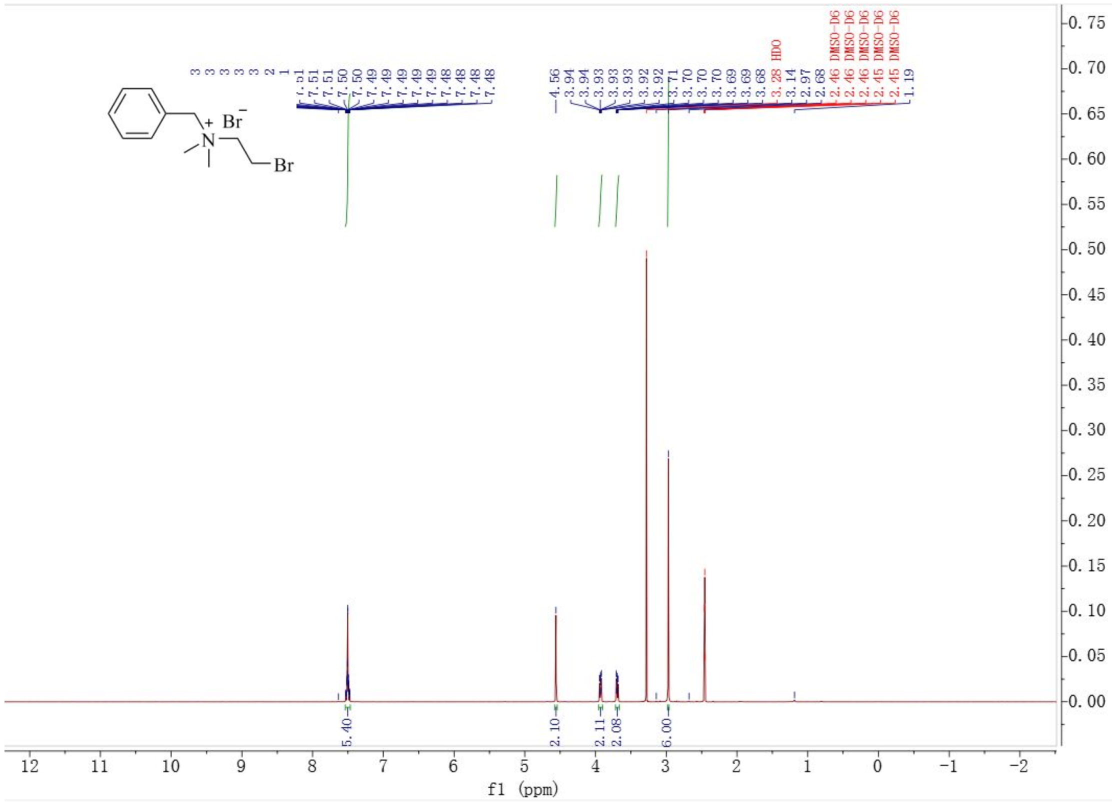

Figure S1. ^{1}H NMR spectrum of **compound 1a** in DMSO-d_6

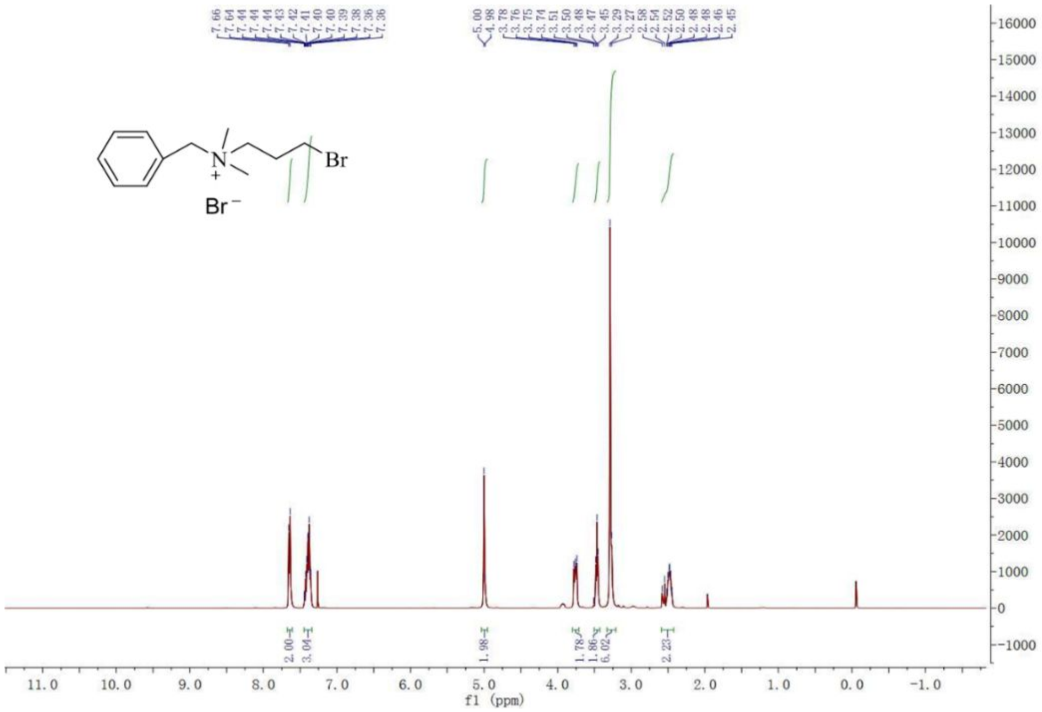

Figure S2. ^{1}H NMR spectrum of **compound 1b** in $CDCl_3$

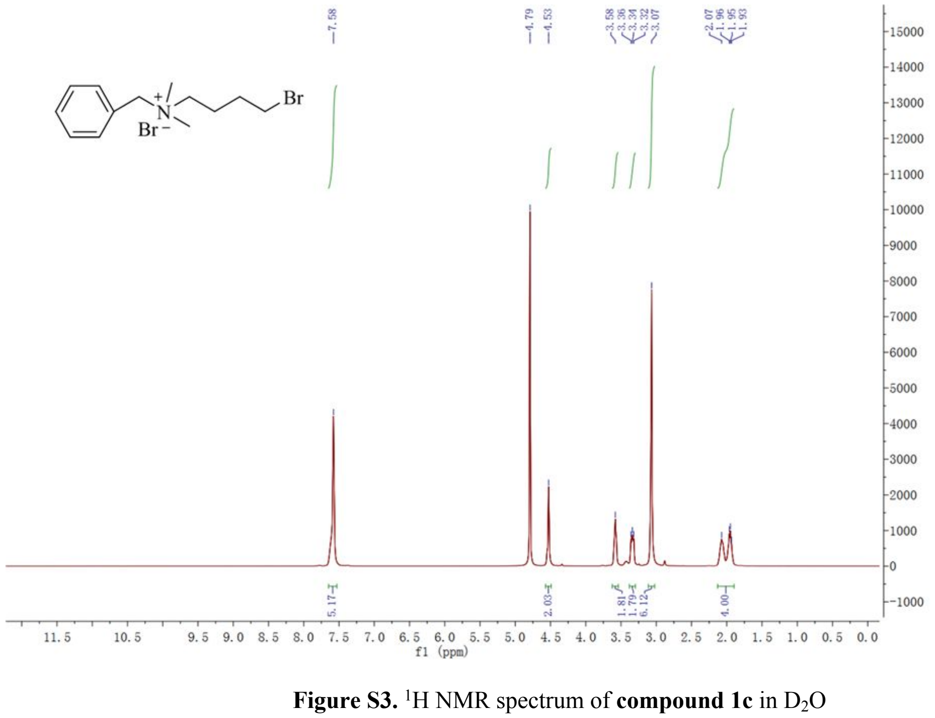

Figure S3. ^{1}H NMR spectrum of **compound 1c** in D_2O

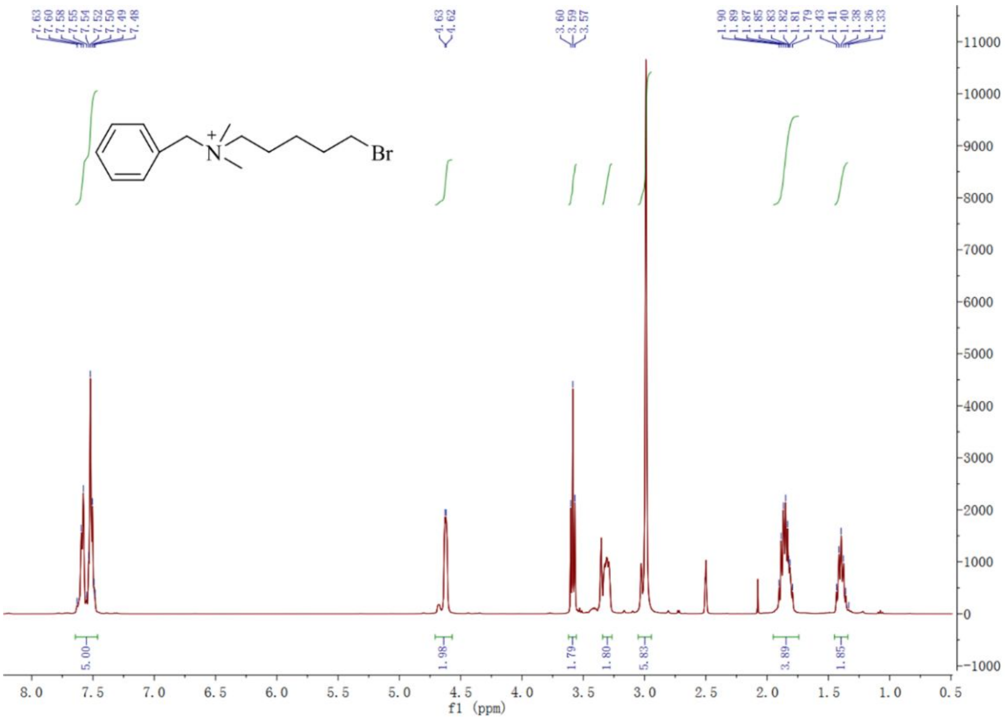

Figure S4. ^{1}H NMR spectrum of **compound 1d** in DMSO-d_6

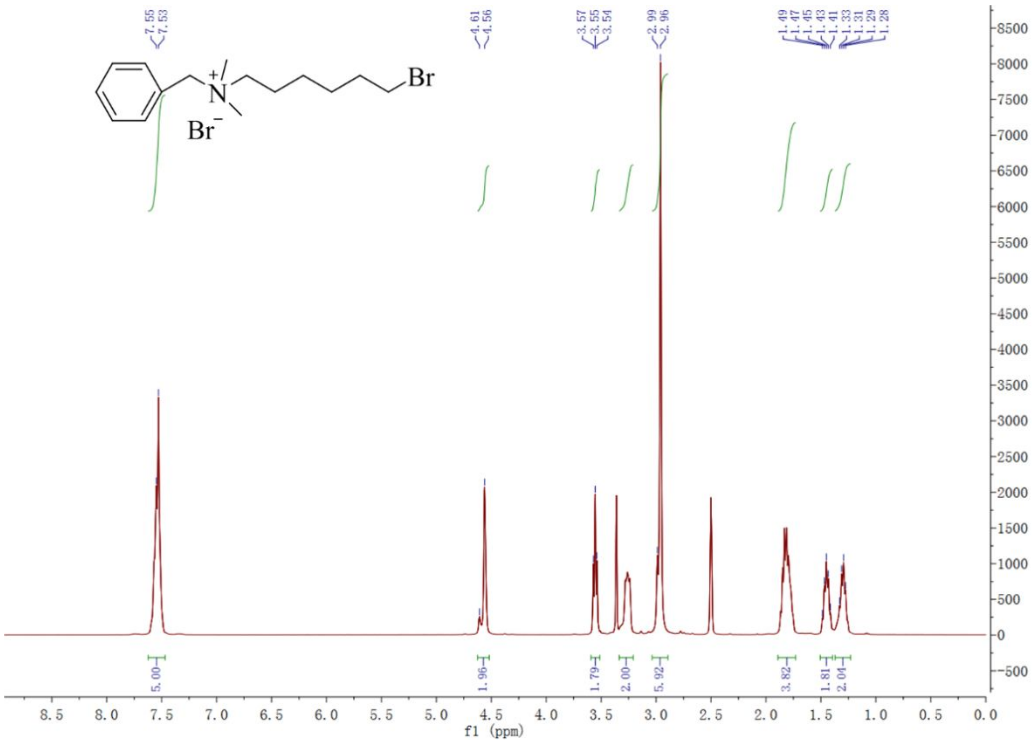

Figure S5. ^{1}H NMR spectrum of **compound 1e** in DMSO-d_6

2. Synthesis of the PAN$_P$F

Dried PANF (1.0 g), *N*,*N*-dimethyl-1, 3-propanediamine (20 mL) and deionized water (10 mL) were added to a three-necked flask. The mixture was stirred and refluxed for 4.5 h. After that, the modified fiber was filtered out and washed with hot water (60-70 °C) until neutral. The fiber was dried overnight at 60 °C under vacuum to give PANpF. The weight gain of PAN$_P$F based on PANF is 24.5%.

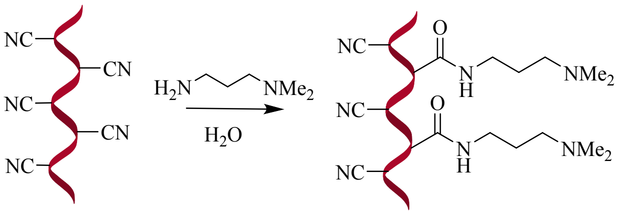

Scheme S2. Synthesis of the PAN$_P$F

3. Synthesis of the PAN$_{QAS-1}$F and PAN$_{QAS-2}$F

Dried PAN$_P$F (1.00 g), benzyl bromide (or bromoethane) 5 mL and ethanol (20 mL) were added to a three-necked flask. The mixture was stirred and refluxed for 4.0 h. After the reaction finished, the functionalized fiber was filtered out and washed with ethanol in a soxhlet extractor to remove unreacted small molecules. After extraction for 12 h, the fiber was filtered out and then was dried overnight at 60 °C under vacuum to give PAN$_{QAS-1}$F (PAN$_{QAS-2}$F). The weight gain of PAN$_{QAS-1}$F (PAN$_{QAS-2}$F) based on PAN$_P$F is 36.5%, and its functionality is 1.63 mmol g^{-1} (the weight gain of PAN$_{QAS-2}$F is 24.1% and the functionality is 1.79 mmol g^{-1}).

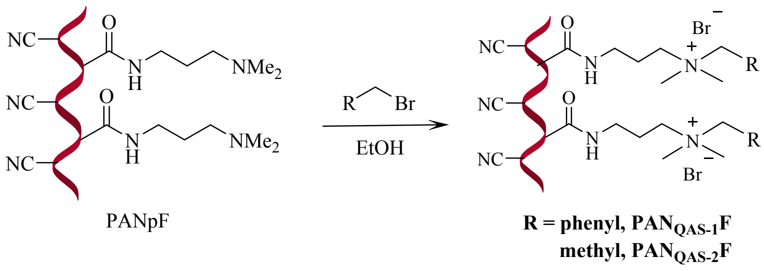

Scheme S3. Synthesis of PAN$_{QAS-1}$F and PAN$_{QAS-2}$F

4. Synthesis of the $PAN_{BQAS-2}F$, $PAN_{BQAS-3}F$, $PAN_{BQAS-4}F$, $PAN_{BQAS-5}F$ and $PAN_{BQAS-6}F$

Dried PAN_PF (1.0 g), **compound 1a-compound 1e** (2 mmol) and ethanol (20mL) were added to a three-necked flask. The mixture was stirred and refluxed for 4.0 h, the fiber was then filtered out and washed with ethanol in a soxhlet extractor to remove unreacted small molecules. After extraction for 12 h, the fiber was dried overnight at 60 °C under vacuum to obtain $PAN_{BQAS-n}Fs$. The weight gain of $PAN_{BQAS-2}F$, $PAN_{BQAS-3}F$, $PAN_{BQAS-4}F$, $PAN_{BQAS-5}F$ and $PAN_{BQAS-6}F$ based on PAN_PF is 54.0%, 73.6%, 62.8%, 64.2%, 40.3%, respectively.

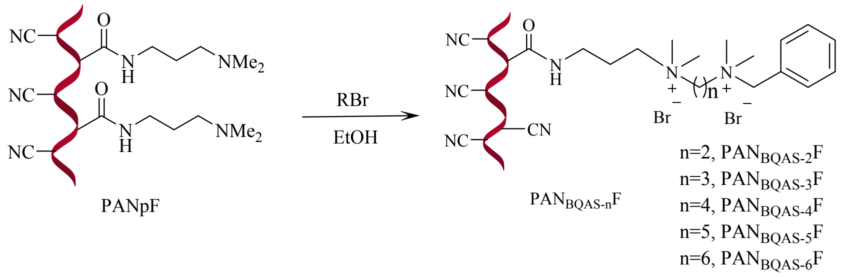

Scheme S4. Synthesis of $PAN_{BQAS-n}Fs$

5. Mixed phenolic adsorption experiment

Dried $PAN_{BQAS-3}F$ (15 mg) was immersed into 40 mL solution (200 mg L^{-1}) and then the mixture was stirred a for 8 h. The concentration of phenol and 2,4-dinitrophenol were determined by UV at 269 nm and 359 nm respectively. The concentration of thiophenolate sodium determined by UV at 263 nm. All initial and final concentrations were calculated by standard curve of UV.

6. Thermogravimetric analysis (TGA)

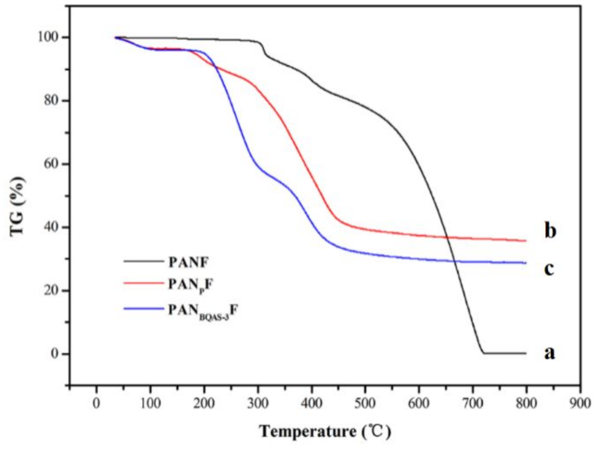

Figure S6. TGA spectra of (a) PANF, (b) PAN_PF and (c) $PAN_{BQAS\text{-}3}F$

7. XRD patterns

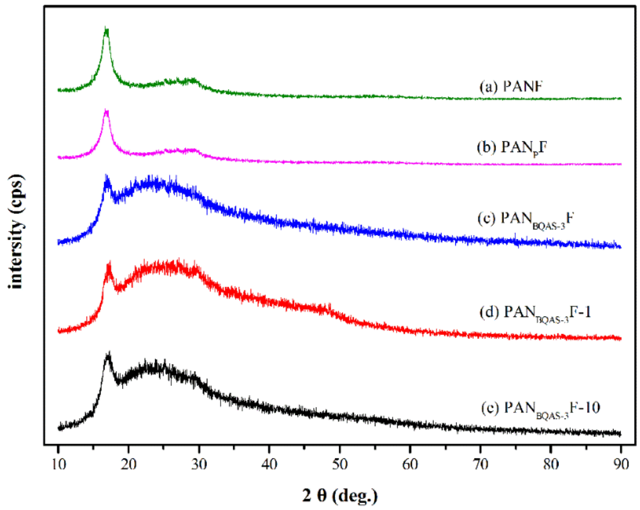

Figure S7. The XRD spectra of the (a) PANF, (b) PANPF, (c) $PAN_{BQAS\text{-}3}F$, (d) $PAN_{BQAS\text{-}3}F$-1, (e) $PAN_{BQAS\text{-}3}F$-10.

8. Effect of $PAN_{BQAS\text{-}3}F$ dosage on adsorption capacity

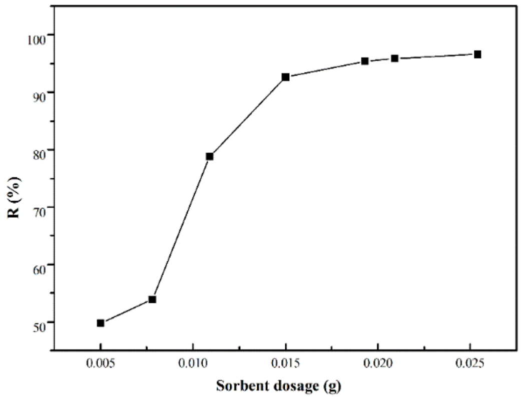

Figure S8. The effect of adsorbent dosage on the adsorption of 2,4-DNP.

9. Effect of solution pH on the adsorption capacity

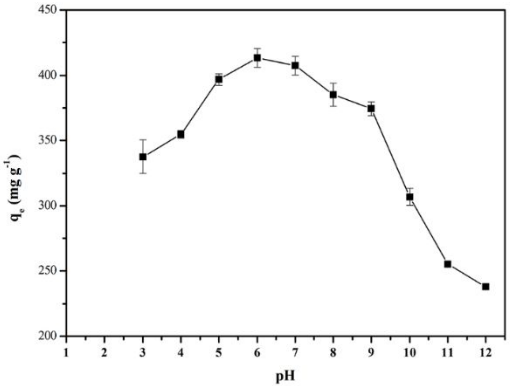

Figure S9. Effect of pH value on the adsorption of 2,4-DNP by $PAN_{BQAS-3}F$

10. Relationship between pH initial and pH final for pHpzc determination

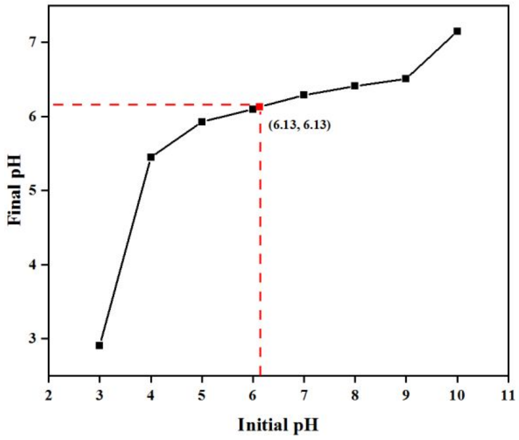

Figure S10. Relationship between initial pH and final pH for pHpzc determination

11. Standard concentration curve of 2,4-dinitrophenol and 4-nitrophenol

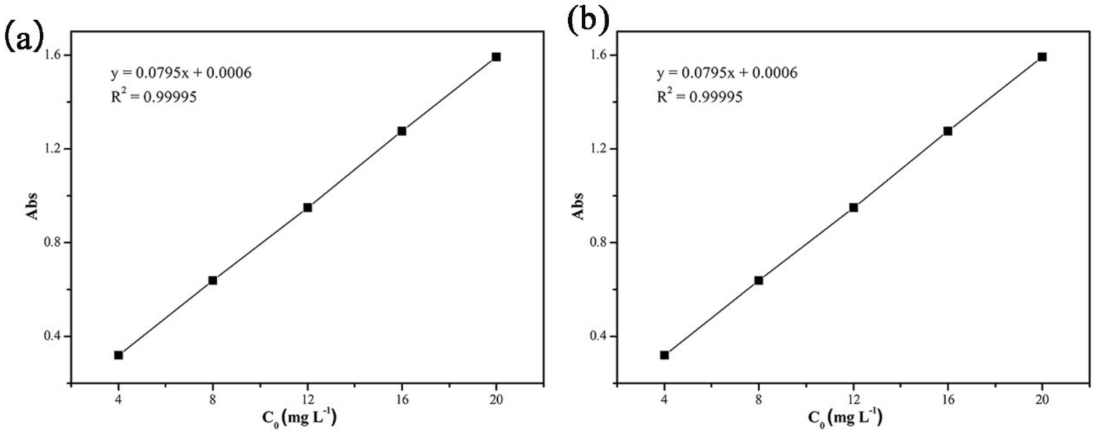

Figure S11. (a) Standard curve of 2,4-dinitrophenol by UV at 359 nm; (b) Standard curve of 4-Nitrophenol by UV at 400nm

12. Pseudo first-order and pseudo second-order kinetics model for the adsorption of 2,4-dinitrophenol by $PAN_{BQAS-3}F$

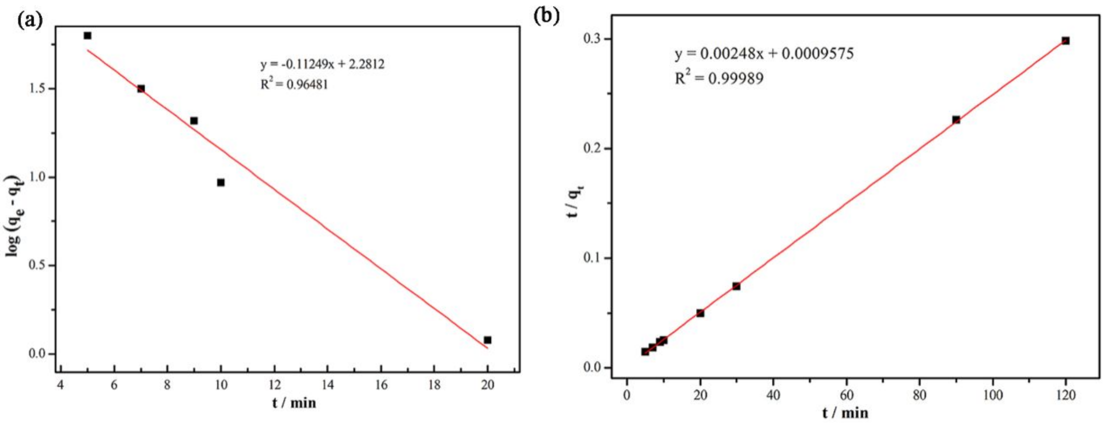

Figure S12. (a) Pseudo first-order plot for 2, 4-dinitrophenol adsorption by $PAN_{BQAS-3}F$; (b) Pseudo second-order plot for 2, 4-dinitrophenol adsorption by $PAN_{BQAS-3}F$

13. Langmuir and Freundlich isotherm plots for the adsorption of 2,4-dinitrophenol by $PAN_{BQAS-3}F$

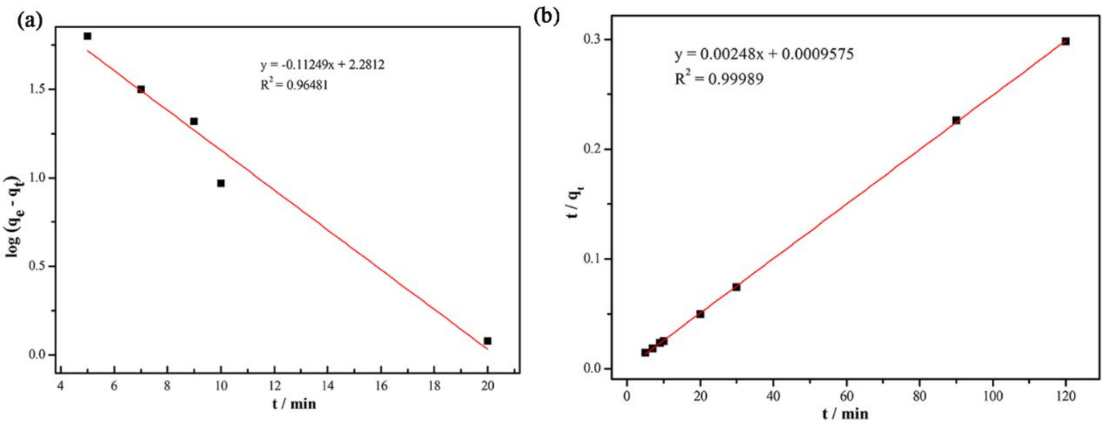

Figure S13. (a) Langmuir isotherm model fitting diagram; (b) Freundlich isotherm fitting diagram

14. Breakthrough curves for 2,4-DNP solutions

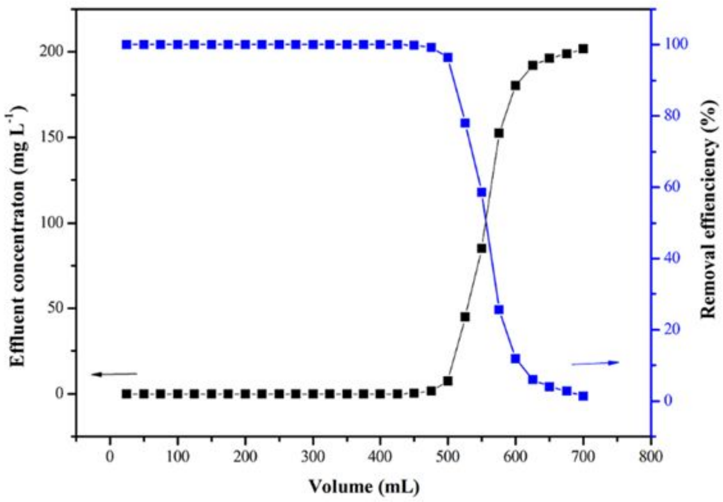

Figure S14. Breakthrough curves for 2,4-DNP solutions

15. Comparison of 2,4-dinitrophenol removal with recently reported adsorbents

Table S1 Compare $PAN_{BQAS-3}F$ with others adsorbents

Entry	Adsorbents	Dosage (g/L)	Concentration (ppm)	Time (min)	q_{max} (mg g^{-1})	Run	Ref.
1	Fe_3O_4@AC-SS	0.02	60	60	43.00	3	3
2	Ionic liquid functionalized polymer（IL-P）	1	73.64	60	56.86	5	4
3	UIO-66	0.4	80	360	144.10	5	5
4	UIO-66-NH_2	0.4	80	360	139.39	5	5
5	char ash	20	40	580	7.55	/	6
6	Titania-silica mixed imidazolium based ionic liquid (Ti-Si-IL)	2	10	30	44.64	/	7
7	SilprP$_3$NImBr	0.3	50	18	91.74	8	8
9	activated carbon fibers	0.1	270	1440	1.49	/	9
10	XG-g-Polyaniline@ZnO Nanocomposite	2	120	120	123.15	5	10
11	Fe_3O_4@AC-SS	2	60	120	43.00	4	11
12	$PAN_{BQAS-3}F$	0.6	200	20	406.00	10	This work

16. Removal of 2,4-dinitrophenol by $PAN_{BQAS-3}F$ under continuous flow condition

Please refer to the video attachment 1.

17. Desorption of 2,4-dinitrophenol by $PAN_{BQAS-3}F$ under continuous flow condition

Please refer to the vedio attachment 2.

18. Reference

(1) Oyervides-Muñoz, E.; Pollet, E.; Ulrich, G.; de Jesús Sosa-Santillán, G.; Avérous, L. Original method for synthesis of chitosan-based antimicrobial agent by quaternary ammonium grafting. *Carbohyd. Polym.* **2017**, *157*, 1922-1932.

(2) Xing, X.; Yang, H.; Tao, M.; Zhang, W. An overwhelmingly selective colorimetric sensor for Ag^+

using a simple modified polyacrylonitrile fiber. *J. Hazard. Mater.* **2015**, *297*, 207-216.

(3) Gopal, K.; Mohd, N. I.; Raoov, M.; Suah, F. B. M.; Yahaya, N.; Zain, N. N. M. Development of a new efficient and economical magnetic sorbent silicone surfactant-based activated carbon for the removal of chloro- and nitro-group phenolic compounds from contaminated water samples. *RSC Adv.* **2019**, *9*, 36915-36930.

(4) Zhu, G.; Cheng, G.; Lu, T.; Cao, Z.; Wang, L.; Li, Q.; Fan, J. An ionic liquid functionalized polymer for simultaneous removal of four phenolic pollutants in real environmental samples. *J. Hazard. Mater.* **2019**, *373*, 347-358.

(5) Lv, G.; Liu, J.; Xiong, Z.; Zhang, Z.; Guan, Z. Selectivity adsorptive mechanism of different nitrophenols on UIO-66 and UIO-66-NH_2 in aqueous solution. *J. Chem. Eng. Data.* **2016**, *61*, 3868-3876.

(6) Magdy, Y. M.; Altaher, H.; ElQada, E. Removal of three nitrophenols from aqueous solutions by adsorption onto char ash: equilibrium and kinetic modeling. *Appl. Water Sci.* **2018**, *8*.

(7) Ismail, N. A.; Bakhshaei, S.; Kamboh, M. A.; Abdul Manan, N. S.; Mohamad, S.; Yilmaz, M. Adsorption of phenols from contaminated water through titania-silica mixed imidazolium based ionic liquid: Equilibrium, kinetic and thermodynamic modeling studies. *J. Macromol. Sci., Part A: Pure Appl.Chem.* **2016**, *53*, 619-628.

(8) Wang, Z.; Ye, C.; Wang, H. Preparation of amino functionalized imidazolium-modified silicas by different coupling agents for removal of 2,4-dinitrophenol from aqueous solutions. *Int. J. Environ. Sci. Te.* **2016**, *13*, 113-124.

(9) Liu, Q.; Zheng, T.; Wang, P.; Jiang, J.; Li, N. Adsorption isotherm, kinetic and mechanism studies of some substituted phenols on activated carbon fibers. *Chem. Eng. J.* **2010**, *157*, 348-356.

(10) Ahmad, R.; Hasan, I. Efficient remediation of an aquatic environment contaminated by Cr(VI) and 2,4-dinitrophenol by XG-g-Polyaniline@ZnO nanocomposite. *J. Chem. Eng. Data.* **2017**, *62*, 1594-1607.

(11) Gopal, K.; Mohd, N. I.; Raoov, M.; Suah, F. B. M.; Yahaya, N.; Zain, N. N. M. Development of a new efficient and economical magnetic sorbent silicone surfactant-based activated carbon for the removal of chloro- and nitro-group phenolic compounds from contaminated water samples. *RSC Adv.* **2019**, *9*, 36915-36930.
